# Isotope analysis of human dental calculus δ^13^CO_3_^2−^: Investigating a potential new proxy for sugar consumption

**DOI:** 10.1002/rcm.9286

**Published:** 2022-03-25

**Authors:** Blessing Chidimuro, Amy Mundorff, Camilla Speller, Anita Radini, Noémie Boudreault, Mary Lucas, Malin Holst, Angela Lamb, Matthew Collins, Michelle Alexander

**Affiliations:** ^1^ Department of Geography and Environmental Science University of Reading Reading UK; ^2^ BioArCh, Department of Archaeology University of York York UK; ^3^ Department of Anthropology University of Tennessee Knoxville Tennessee USA; ^4^ Department of Anthropology University of British Columbia Vancouver BC Canada; ^5^ Department of Archaeology Max Planck Institute for the Science of Human History Jena Germany; ^6^ York Osteoarchaeology Ltd, Bishop Wilton York UK; ^7^ National Environmental Isotope Facility, British Geological Survey Keyworth UK; ^8^ Natural History Museum University of Copenhagen Denmark; ^9^ Department of Archaeology University of Cambridge Cambridge UK

## Abstract

**Rationale:**

Dental calculus (mineralised dental plaque) is composed primarily of hydroxyapatite. We hypothesise that the carbonate component of dental calculus will reflect the isotopic composition of ingested simple carbohydrates. Therefore, dental calculus carbonates may be an indicator for sugar consumption, and an alternative to bone carbonate in isotopic palaeodiet studies.

**Methods:**

We utilised Fourier transform infrared attenuated total reflectance analysis to characterise the composition and crystallisation of bone and dental calculus before isotope analysis of carbonate. Using a Sercon 20‐22 mass spectrometer coupled with a Sercon GSL sample preparation system and an IsoPrime 100 dual inlet mass spectrometer plus Multiprep device to measure carbon, we tested the potential of dental calculus carbonate to identify C_4_ resources in diet through analysis of δ^13^C values in paired bone, calculus and teeth mineral samples.

**Results:**

The modern population shows higher δ^13^C values in all three tissue carbonates compared to both archaeological populations. Clear differences in dental calculus δ^13^C values are observed between the modern and archaeological individuals suggesting potential for utilising dental calculus in isotope palaeodiet studies. The offset between dental calculus and either bone or enamel carbonate δ^13^C values is large and consistent in direction, with no consistent offset between the δ^13^C values for the three tissues per individual.

**Conclusions:**

Our results support dental calculus carbonate as a new biomaterial to identify C_4_ sugar through isotope analysis. Greater carbon fractionation in the mouth is likely due to the complex formation of dental calculus as a mineralized biofilm, which results in consistently high δ^13^C values compared to bone and enamel.

## 1 INTRODUCTION

Investigations into past diets frequently draw upon direct isotopic measurements of the surviving body tissues of consumers. Bone and teeth most commonly survive in archaeological contexts but soft tissues (e.g. skin), hair and fingernails can also provide dietary information if they are preserved. Dental calculus (mineralised dental plaque) has recently received attention for its potential to reveal aspects of past diets, primarily through the analysis of biomolecules – proteins, DNA^1^, ^2^ – and micro‐debris trapped within the mineral matrix.^3^ Only a few studies have explored the potential for isotopic analysis of dental calculus but they have mainly analysed bulk carbon (δ^13^C) and nitrogen (δ^15^N) isotope compositions of the organic matrix with mixed results.^4^, ^5^, ^6^, ^7^, ^8^ One recent study specifically targeted the inorganic fraction of calculus, suggesting that this component may have more validity as a palaeodiet proxy; however, the study was limited in terms of the methodology as well as the small number of samples analysed.^8^ The present study goes further to explicitly explore the potential utility of δ^13^C in dental calculus mineral as a dietary proxy for identifying C_4_ sugar (maize/cane). Building on previous studies applying Fourier transform infrared (FTIR) spectroscopy to characterise dental calculus composition,^9^, ^10^ we apply this analysis to systematically characterise the composition and crystallisation of dental calculus. This study presents the first analysis and interpretation of dental calculus FTIR results as an assessment of diagenesis in line with what has been previously undertaken for bone and enamel carbonate. It goes on to present the first comparison of dental calculus carbonate with both bone and enamel carbonate to determine whether dental calculus carbonate is a suitable substrate for identifying C_4_ resource consumption. In addition, the results obtained here are used to determine if there is any intra‐individual variation among tissues. This analysis is notable for analysing material from archaeological populations as well as modern individuals, with the latter rarely incorporated into archaeological studies.

The current understanding of diet in palaeodietary studies utilising stable isotopes (δ^13^C, δ^15^N) is mainly based on the analysis of bone and dentine collagen (δ^13^C, δ^15^N) as well as enamel and bone carbonate (δ^13^C).^11^, ^12^ Bone and dentine collagen δ^13^C values represent the protein sources in the diet, but a proportion of collagen can be synthesised from lipids or carbohydrates. Bone and enamel carbonate δ^13^C values, however, reflect whole diets including carbohydrates, lipids and proteins.^13^ Palaeodietary reconstruction using carbon isotope values is useful in distinguishing between the consumption of C_3_ (low δ^13^C value) and C_4_ (high δ^13^C value) terrestrial resources as well as between marine (high δ^13^C value) and terrestrial (low δ^13^C value) foods. Nitrogen isotope values provide information on the main protein sources of diet, for example, differentiating between plant‐rich protein and animal‐rich protein diets.^14^

Previously, C_4_ cane sugar consumption has only been detected under specific circumstances, such as when sugarcane is an indigenous crop^15^ or inferred where sugarcane may have been used as animal fodder, in which case the C_4_ signal is acquired by herbivores.^16^ Neither maize nor cane sugar would have been available to the medieval populations considered here.^17^ The post‐medieval period between the 17th and 19th centuries, however, saw an increase in the consumption of cane sugar in England due to Britain's colonisation of the West Indies in the 17th century.^18^, ^19^ Although maize was available in post‐medieval England, historical sources indicate that it was considered to be suitable for animal fodder or famine relief food, particularly for the Irish poor.^20^, ^21^ However, a recent analysis of dental calculus micro‐debris of 36 individuals from the middle‐class Cross Street population revealed that two individuals had the presence of maize (not included in this study).^3^ Maize is therefore another possible source of C_4_ carbohydrate for the post‐medieval population; however, sugar will have been more ubiquitous. Although hypothesised in some bone collagen isotope studies of post‐medieval populations in England,^22^, ^23^, ^24^, ^25^ the consumption of cane sugar has been hard to detect despite it being a key element of the dietary staple. In this study, it is hypothesised that dental calculus carbonate could offer a novel and more sensitive way of identifying cane sugar/maize than the traditional isotopic methods focusing on bone.

### 1.1 Dental calculus and potential for isotopic analysis

Calculus is frequently preserved in archaeological contexts and has been found to survive on the teeth of late Pliocene hominins^26^ and Miocene apes dating up to 8 to 12 million years ago.^27^ Dental calculus results from the calcification of plaque biofilms that accumulate and mineralise during life; however, the mechanism and rate by which dental calculus forms are still not completely understood.^28^ Of the two types of dental calculus, supragingival and subgingival, the present study attempted to target supragingival dental calculus for all analyses.

Plaque formation on the supragingival surface of teeth begins when microorganisms, overwhelmingly bacteria, colonise the pellicle on the tooth surface. These bacteria obtain their nutrients primarily from the amino acids, proteins, glycoproteins and peptides from saliva to grow, confluence and produce a biofilm.^29^ During the production of the biofilm, extracellular polymer synthesis occurs resulting in glucans and fructans from sucrose (refined carbohydrate) metabolism becoming part of the plaque matrix.^29^, ^30^ Plaque may begin to harden after about ten days to form dental calculus which then builds up over time. Depending on an individual's hygiene, diet and lifestyle, the deposition of plaque begins soon after tooth eruption, and the quantity increases over time, ceasing at death when the production of saliva stops. Mineralisation of dental plaque only occurs in the presence of saliva when an individual is alive.^31^, ^32^ Calculus formation is facilitated by alkaline conditions in the mouth which in turn increases the precipitation of minerals from the saliva.^33^ Mineralisation also depends on the food being consumed, salivary flow, oral hygiene and the genetics of the individual.^34^ The mineral composition of calculus varies according to the concentration of calcium and phosphorus, the presence of calcification promoters such as urea, fluoride and silicon^35^ and presumably also by the bicarbonate composition in the saliva – itself a function of the rate of carbohydrate metabolism.^30^

Dental calculus is composed of about 20% organic and 80% inorganic constituents.^29^ Calculus deposits generally contain the inorganic mineral calcium phosphate: crystalline forms of hydroxyapatite, octacalcium phosphate and whitlockite in varying quantities, with hydroxyapatite usually being the most abundant (*ca* 58%), which has high levels of carbonate.^36^ The organic matrix contains trapped proteins, glycoproteins, plant fibres, lipids and carbohydrates.^29^ Unlike the carbonate component of bone and enamel, which derives from blood bicarbonate,^37^ supragingival calculus derives its carbonate from the precipitated bicarbonate of salivary fluids.^38^ Experiments have indicated that salivary fluids derive bicarbonate from two sources in the body: (i) transfer from the blood and (ii) bicarbonate that is produced in the cells of the salivary glands.^39^ The salivary gland bicarbonate is from carbon dioxide (CO_2_) resulting from the secretory activity of the salivary gland cell (based on the equilibrium CO_2_ + H_2_O ⇌ HCO_3_^−^ + H^+^) during the action of microbes when metabolising carbohydrates in the mouth. The concentration of bicarbonate is greatly increased during food intake and mastication.^30^, ^40^

As previously mentioned, dental calculus formation is, in part, linked to the consumption of high levels of carbohydrates due to sugars that are eventually converted to CO_2_.^29^, ^32^ Similarly, consumption of carbohydrate‐containing foods has been associated with the formation of dental caries. It has been determined that dental caries develops due to a dissolution of the enamel by the action of acids that are produced in the mouth by the fermentation of dietary carbohydrates by oral bacteria, particularly *Streptococcus mutans*.^41^ As is evident by the link between sugar consumption and dental caries, oral microbial communities preferentially metabolise the most bioavailable components of dietary carbohydrate.^42^, ^43^ Therefore, it is likely that ^13^C‐enriched CO_2_ generated from microbial carbohydrate metabolism in the mouth and from the blood via the salivary glands is incorporated into calculus carbonate. The hypothesis, therefore, is that calculus carbonate will be more responsive than bone apatite to the consumption of C_4_ cane sugar/maize in post‐medieval England, which has been difficult to identify using other isotope methods.

To fully assess the potential for dental calculus in palaeodietary studies, and particularly for the possibility of determining C_4_ cane sugar/maize consumption for post‐medieval England, isotope analysis on dental calculus, bone and enamel obtained from the same individuals was conducted in a broad range of populations.

## 2 MATERIALS AND METHODS

### 2.1 Materials

Bone, enamel and dental calculus samples were collected from 57 individuals for bone collagen, bone carbonate, enamel carbonate and calculus carbonate analysis. The three different populations sampled for this study were as follows: (i) 22 individuals from medieval burial populations from England, including Southwell Cemetery, Nottinghamshire (SCN), St Peter's Cemetery, Leicester (SPL) and Nun's Field, Chester (NFC) dating between the 7th and 15th centuries CE, (ii) 15 post‐medieval individuals buried in the Cross Street Chapel cemetery, Manchester (CSM), England, dating to the 18th–19th centuries CE and (iii) 20 modern individuals (FAC), who died between 1996 and 2016, from the William M. Bass Donated Skeletal Collection housed in the Department of Anthropology, University of Tennessee, USA. Additional information on the ethical considerations and treatment conditions for the modern materials can be found in the supporting information.

For medieval and post‐medieval individuals, the first priority was to sample individuals with dental calculus. Ribs were preferentially sampled; however, where ribs were not available, long bones were sampled. In the case of Southwell and St Peter's Cemetery, only mandibles were available to sample. For the modern individuals, the majority of samples were pedal phalanges, two were 12th ribs and one was a hand phalanx. These samples were selected for two reasons. First, these particular elements are numerous and relatively non‐diagnostic compared to other elements, and therefore preferable for destructive analysis in order to preserve the majority of the skeleton for future research. Second, in a large study testing all the skeletal elements from numerous individuals specifically from the Bass Collection,^44^ these elements consistently yielded full STR profiles, indicating good biomolecular preservation.

Previous isotope analysis has revealed that medieval populations consumed C_3_ terrestrial resources.^45^ Similarly, diet for post‐medieval individuals has been found to have been dominated by C_3_ terrestrial resources, although C_4_ cane sugar/maize is known to have been part of their diet or available.^25^, ^46^ Finally, the modern population represents North American individuals, who almost certainly consumed an abundance of C_4_ sugar and maize. Currently, the intake of sugar refined from C_4_ plants such as corn and cane syrup makes up to 78% of the sugar consumed in the United States and forms around 16% of the total calories consumed; however, in some cases it exceeds 35%.^47^ The modern group, therefore, provides a control for diets rich in C_4_ sources.

### 2.2 Methods

#### 2.2.1 FTIR with attenuated total reflectance (ATR)

Sample preparation and analysis of 57 bone and 52 dental calculus samples were executed according to the Kontopoulos et al^48^ method. We carried out FTIR‐ATR analysis before acetic acid treatment. The use of ATR during FTIR analysis has the advantage of being generally insensitive to sample thickness.^49^ Bones were cleaned prior to analysis using a sterile scalpel blade and dental calculus samples were rinsed with deionised water to remove dirt and contaminating material. Once cleaned, the samples were ground using an agate mortar and pestle. The powdered samples were then sieved through Endecotts woven stainless steel mesh sieves with an aperture size of 20 and 50 μm so that only grains between 20 and 50 μm particle size would be collected. Spectral analyses were performed using OPUS software (Bruker). Spectra were collected in 144 scans, in the 400–4000 cm^−1^ wavenumber range, with a spectral resolution of 4 cm^−1^ and zero‐filling factor of 4. Each sample was measured in triplicate and *ca* 2–3 mg of powder was pressed onto the diamond crystal and measured. We cleaned the instrument's crystal and arm's tip with tissue paper soaked in propanol before each measurement. Baseline correction and spectra normalisation were carried out using OPUS software as reported in Kontopoulos et al.^48^ We followed the established bone FTIR analysis method for dental calculus samples as there is no agreed standard for the latter yet. We used two quality parameters, the infrared splitting factor (IRSF) and the carbonate‐to‐phosphate ratios (C/P), to assess our samples. IRSF is used to evaluate the crystallinity (structural order) within the mineral component of the bone while the C/P ratio is a measure of diagenesis that reflects the changes to the carbonate in bioapatite crystals relative to the phosphate content ratio in a bone sample. We calculated IRSF indices following the method of Weiner and Bar‐Yosef^50^ that measures the heights of the double peaks of the phosphate antisymmetric bending frequency between 550 and 650 cm^−1^,^51^ divided by the trough between them:

$$\text{IRSF}=\left[{\left(v_{4}{{\mathrm{PO}}_{4}}^{3-}\right)}_{\mathrm{ht}}+{\left(v_{4}{{\mathrm{PO}}_{4}}^{3-}\right)}_{\mathrm{ht}}\right]/{\text{trough}}_{\mathrm{ht}}$$

The C/P ratio was estimated using the method of Wright and Schwarcz^52^ by dividing the main *v*_3_ carbonate peak height with the main *v*_3_ phosphate vibrational band:

$$C/P={\left(v_{3}{{\mathrm{CO}}_{3}}^{2-}\right)}_{\mathrm{ht}}/{\left(v_{3}{{\mathrm{PO}}_{4}}^{3-}\right)}_{\mathrm{ht}}$$

For bone, the peaks were at 565 cm^−1^ (*v*_4_PO_4_^3−^), 605 cm^−1^ (*v*_4_PO_4_^3−^), 1035 cm^−1^ (*v*_3_PO_4_^3−^) and 1035 cm^−1^ (*v*_3_CO_3_^2−^). Therefore, IRSF and C/P ratio were calculated as follows:

$$\text{IRSF}=\left[565_{\mathrm{ht}}+605_{\mathrm{ht}}\right]/590_{\mathrm{ht}}\;\text{and}\;C/P=1415_{\mathrm{ht}}/1035_{\mathrm{ht}}$$

For dental calculus, the absorption peaks used followed the measurements of Hayashizaki et al,^9^ i.e. 580 cm^−1^ (*v*_4_PO_4_^3−^), 600 cm^−1^ (*v*_4_PO_4_^3−^), 595 cm^−1^ (*v*_3_PO_4_^3−^) and 870 cm^−1^ (*v*_3_CO_3_^2−^), resulting in IRSF and C/P ratios being calculated as follows:

$$C/P=870_{\mathrm{ht}}/595_{\mathrm{ht}}\;\text{and IRSF}=\left[580_{\mathrm{ht}}+600_{\mathrm{ht}}\right]/590_{\mathrm{ht}}$$

We also utilised the mean values of a modern bovine bone as a reference throughout.

#### 2.2.2 Bone collagen extraction

Lipids were removed from all modern material prior to collagen and carbonate analysis following Colonese et al.^53^ The samples were rinsed six times in a 2:1 dichloromethane–methanol solvent solution (3 × 2 mL), ultrasonicated for 15 min and centrifuged (850*g*) for 10 min. They were then rinsed with deionised water and dried at room temperature. Collagen extraction from the 57 bone samples followed the Longin^54^ method modified by Brown et al.^55^ Each bone sample was cleaned using a scalpel to remove contaminants from the outer layer of bone. Following this, bone chunks of *ca* 300–500 mg were demineralised in 8 mL of 0.6 M hydrochloric acid (HCl), agitated twice daily. The acid was changed every two days until demineralisation was complete. Next, the supernatant was removed, and the samples were rinsed thrice using deionised water and then gelatinised using HCl (pH 3) at 80°C for 48 h. Next, the supernatant liquid containing the collagen was filtered using Ezee™ filters to remove unwanted particulate matter from the collagen solution and was then frozen for a minimum of 12 h at −20°C before being freeze‐dried for 48 h. Collagen yields were estimated by dividing the collagen mass after filtration by the original bone mass after cleaning.

#### 2.2.3 Bone preparation for carbonate analysis

Bone carbonate analysis followed a procedure adapted from Snoeck and Pellegrini^56^ and Pellegrini and Snoeck.^57^ The bones were cleaned using a sterile scalpel blade to remove dirt and contaminating material. The cleaned bones were crushed using an agate mortar and pestle. Approximately 7.5 mg of bone powder was required for each sample. To remove secondary minerals from the samples, the weighed samples were dissolved in 15 mL of calcium acetate ((CH_3_COO)_2_Ca) buffered 1 M solution (ratio 1:2) and placed on a roller rocker for about 30 min. After treatment, the samples were rinsed six times with deionised water, placed in a freezer for 24 h and freeze‐dried for 24 h to remove all the water and isolate the apatite. Centrifuge tubes (15 mL) containing the treated samples were reweighed and the mass loss generated by the treatment was measured by subtracting the original weight of the tube.

#### 2.2.4 Enamel preparation for carbonate analysis

Enamel carbonate preparation followed the method described in Miller et al.^58^ We separated a section of enamel, approximately 3 mm wide that spanned from the cervical margin to the cusp, from the tooth crown. In order to minimise contamination, we cleaned all tools prior to use and between samples. All surfaces of the enamel samples were lightly drilled with an abrasive drill bit, set at the lowest speed to avoid heating. Any evident cracks, as well as cut surfaces, were lightly drilled. Each enamel chip was placed in a 2 mL Eppendorf vial with deionised water and sonicated for 3 min to remove any fine powder. In cases where water remained cloudy after sonication, repeat washes were performed. Enamel was then finely ground using an agate mortar and pestle to a particle size of less than 50 μm. Possible diagenetic contaminants were removed by soaking the enamel powder in 0.1 mL of 0.1 M acetic acid per 1 mg of enamel and agitated for 10 min. The acid was removed by repeated rinsing in distilled water five times and centrifuging for 2 min at 13 700*g* between rinses. We froze the samples for 24 h and then freeze‐dried them for 24 h to remove all the water and isolate the apatite. The tubes containing the treated samples were reweighed and the mass loss generated by the treatment was measured by subtracting the original weight of the tube.

#### 2.2.5 Dental calculus preparation for carbonate analysis

Methods of separating carbonate from calculus prior to isotope analysis have not been as extensively explored as they have been for bone and enamel. Therefore, our methods followed the only existing published protocols in order to facilitate comparisons and we used the methods outlined in Price et al^8^ with modifications from Crisp et al^59^ to enhance the removal of the organic component of dental calculus. Before carbonate analysis, the optimal time for the calculus to be submerged in NaClO (bleach) was checked using the protocol of Crisp et al.^59^ The bleach was expected to oxidise amino acids rendering them unavailable for analysis. The study of Crisp et al^59^ noted that ostrich eggshells coarse grain (500–1000 μm) free amino acid samples took a long time to demineralise, probably due to the reduced surface area to volume ratio; therefore they decided not to use powdered particles >500 μm in size in further experiments.

In light of the preceding recommendations by Crisp et al,^59^ for this study, the dental calculus samples were first washed in deionised water four times in order to remove any soil loosely adhering to them. The clean calculus was left to dry at room temperature and then powdered with an agate pestle and mortar to <500 μm. Since dental calculus is much richer in organics than shells,^60^, ^61^ considering that for powdered shells, after 72 h, all amino acids accessible by 50 mL of 12% (w/v) NaClO had been removed, the powdered samples in this study were submerged in 50 mL of 12% (w/v) NaClO per milligram of sample for 96 h. The samples were agitated every 24 h to ensure that the whole calculus matrix was exposed to the bleach. The bleach was removed by pipette and spotted onto coloured tissue paper to test if it was still active. The dental calculus was then washed five times in distilled water, with a sixth wash with HPLC‐grade methanol to reduce any leftover bleach and then air‐dried overnight. To extract the mineral in dental calculus, *ca* 1.5 mg of each bleached sample was reacted with 0.2 mL per 5 mg of (CH_3_COO)_2_Ca buffered 1 M solution (ratio 1:2; pH ~ 4.5) and placed on a roller rocker for about 40 min to remove exogenous carbonates. The samples were cleaned with distilled water four times and dried at 40°C.

### 2.3 Analytical measurements

All δ^13^C and δ^15^N ratios are expressed using the delta notation (δ) in parts per thousand (‰) relative to international standards, VPDB for δ^13^C and atmospheric N_2_ (AIR) for δ^15^N, using the following equation:

$$\delta^{i}E_{\text{sample}}=\frac{\left(i_{E}/j_{E}\right)\text{sample}-\left(i_{E}/j_{E}\right)\text{reference}}{\left(\frac{i_{E}}{j_{E}}\right)\text{reference}}$$

where ^*i*^ *E* and ^*j*^ *E* denote the heavier and lighter isotopes, respectively.^62^

#### 2.3.1 Bone collagen

Approximately 0.5 mg of freeze‐dried retentate was weighed out into 4 mm × 3.2 mm tin capsules and combusted alongside international standards in an elemental analyser/isotope ratio mass spectrometer: a Sercon 20‐22 mass spectrometer coupled with a Sercon GSL sample preparation system module at BioArch, University of York. All samples were analysed in duplicate. The duplicate sample 1*σ* reproducibility was <0.2‰ for both δ^15^N and δ^13^C values. Accuracy for bone collagen was determined at the University of York by measurements of international standard reference materials within each analytical run. These were: IAEA 600, δ^13^C_raw_ = −27.65 ± 0.09‰, δ^13^C_true_ = −27.77 ± 0.04‰, δ^15^N_raw_ = 0.92 ± 0.21‰, δ^15^N_true_ = 1 ± 0.2‰; IAEA N2, δ^15^N_raw_ = 20.35 ± 0.13‰, δ^15^N_true_ = 20.3 ± 0.2‰; IA Cane, δ^13^C_raw_ = −11.77 ± 0.09‰, δ^13^C_true_ = −11.64 ± 0.03‰. The overall uncertainties on the measurements of each sample were calculated based on the method of Kragten^63^ by combining uncertainties in the values of the international reference materials and those determined from repeated measurements of samples and reference materials. These are expressed as one standard deviation. The maximum uncertainty for all samples across all runs was <0.2‰ for both δ^13^C and δ^15^N values. In addition, a homogenised bovine bone extracted and analysed within the same batch as the samples produced the following average values: δ^13^C = −23.01 ± 0.13‰; δ^15^N = 6.21 ± 0.44‰. This was comparable to the overall mean value from 50 separate extracts of this bone sample, which produced values of δ^13^C = −22.97 ± 0.19‰ and δ^15^N = 6.19 ± 0.30‰.

#### 2.3.2 Bone, enamel and dental calculus carbonates

All carbonate analysis was carried out using an IsoPrime 100 dual inlet mass spectrometer plus Multiprep device at the Natural Environment Research Council's National Environmental Isotope Facility (Keyworth, Nottingham, UK). Approximately 50–100 μg of each sample was loaded into glass vials and sealed with septa. The vials were evacuated, and anhydrous phosphoric acid was delivered to the carbonate at 90°C. The evolved CO_2_ was collected for 15 min (from the point of acid delivery), cryogenically cleaned and passed to the mass spectrometer. Accuracy for bone, enamel and dental calculus carbonates was determined by measurements of the Keyworth Carrara Marble (KCM), the laboratory's carbonate reference material (average KCM δ^13^C = 2.00 ± 0.02‰), calibrated to NBS19 and NBS18 certified reference material. Accuracy or systematic error (u [bias]) was determined to be ± 0.01 for δ^13^C values on the basis of the difference between the observed and known δ^13^C values of the check standard KCM and the long‐term standard deviation of this check standard. The total analytical uncertainty was estimated to be ± 0.11‰ for the carbonate analysis calculated using the method recommended in Szpak et al^64^ by combining uncertainties in the values of the laboratory's carbonate reference material and those determined from repeated measurements of unknown samples. In addition, three homogenised bovine bones extracted and analysed within the same batch as the samples produced the δ^13^C average values of −16.01 ± 0.08‰.

### 2.4 Suess effect correction

All the modern tissue δ^13^C values were corrected for the Suess effect (Table S4) which is defined as the global decrease of ^14^C and ^13^C relative to ^12^C in atmospheric CO_2_ which occurred primarily due to fossil fuel burning since the Industrial Revolution.^65^ A time‐dependent correction per year to each sample was applied according to Graven et al,^65^ normalised up to 2015. For all individuals who died after 2015, the 2015 normalisation was utilised.

### 2.5 Statistical analysis

Statistical analysis was carried out using R statistics,^66^ PAST statistics^67^ and IBM SPSS statistics version 26.^68^ Non‐parametric statistics were used to compare isotope values among the population groups because of the non‐normal distribution of some data as indicated by Kolmogorov–Smirnov and Shapiro–Wilk tests. The tests were executed using the non‐parametric equivalent of a one‐way analysis of variance and the non‐parametric equivalent of the independent *T*‐test, the Kruskal–Wallis test and the Mann–Whitney test, respectively.

## 3 RESULTS AND DISCUSSION

In total, 57 samples, namely modern (*n* = 20), medieval (*n* = 22) and post‐medieval (*n* = 15), were subjected to bone collagen, bone carbonate, enamel carbonate and calculus carbonate isotope analysis. FTIR‐ATR analysis was performed on 57 bones and 52 dental calculus samples. Tooth enamel is generally assumed to be resistant to diagenetic alteration because of its structure,^69^, ^70^ and since there was not enough material for FTIR analysis as well as isotope analysis, FTIR‐ATR analysis on enamel samples was not performed. Raw isotope data and information for all remains are presented in Table S1.

### 3.1 FTIR‐ATR analysis of bone and dental calculus

**FIGURE 1:**
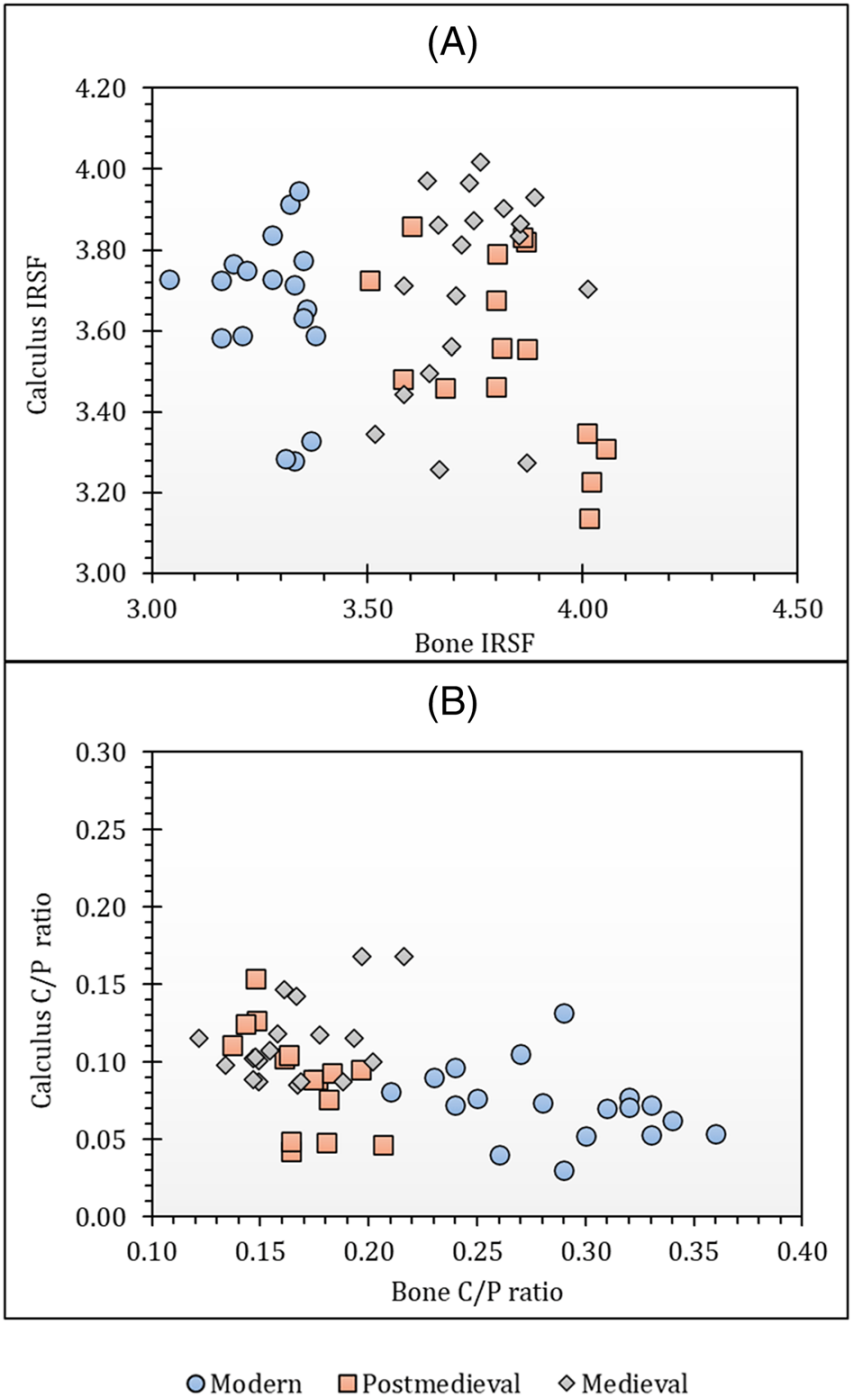
Comparisons between (A) bone IRSF and dental calculus IRSF and (B) bone C/P ratio and calculus C/P ratio [Color figure can be viewed at wileyonlinelibrary.com]

**FIGURE 2:**
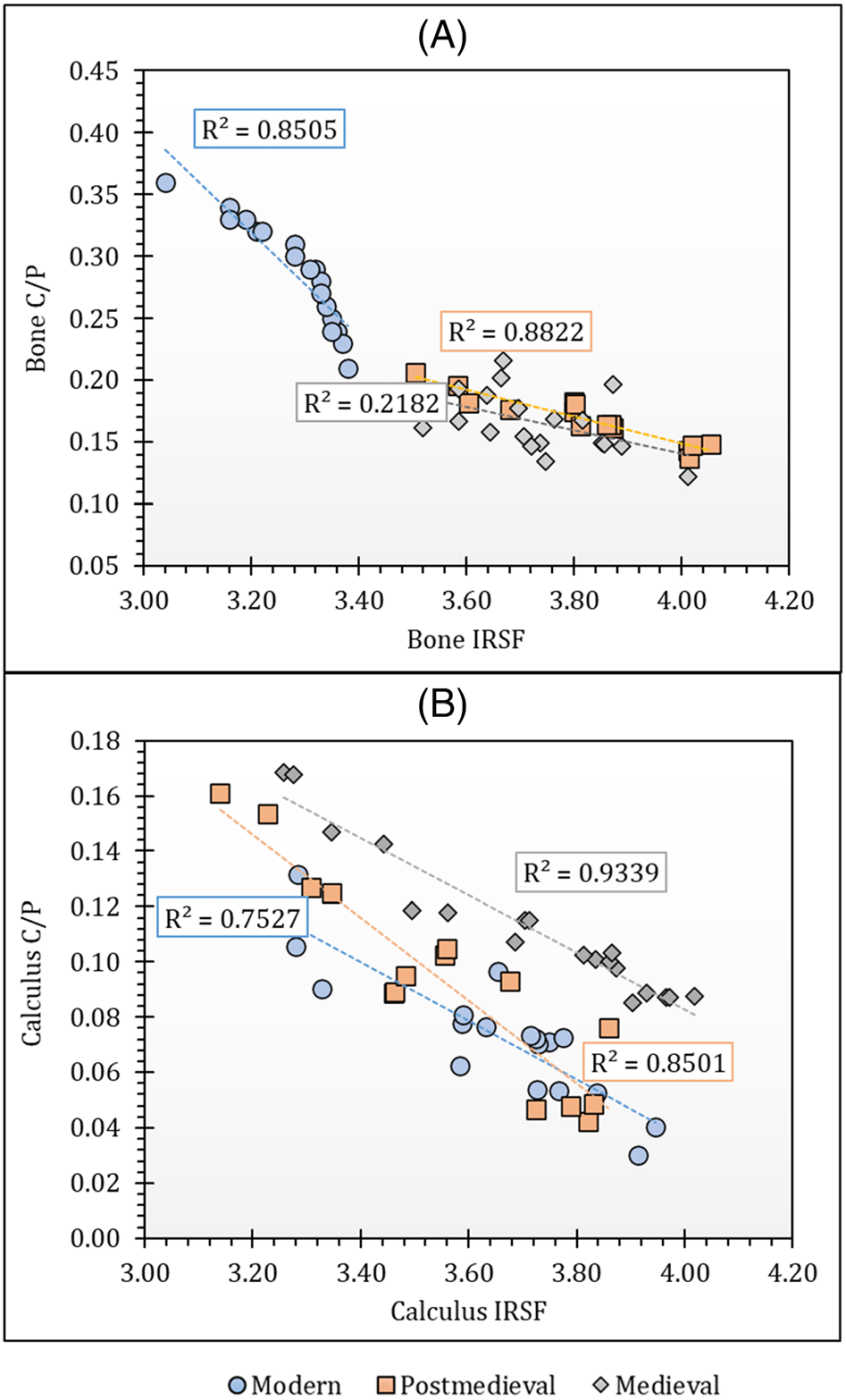
Plots of (A) bone IRSF versus bone C/P ratio and (B) calculus IRSF versus calculus C/P ratio [Color figure can be viewed at wileyonlinelibrary.com]

Diagenesis in bones and dental calculus was assessed using FTIR‐ATR, but dental calculus was insufficient to use for both FTIR and isotope analysis in five of the individuals studied. FTIR data from all sites in this study are provided in Table S3 and plotted in Figures 1 and 2. Analysis and interpretation of FTIR results for dental calculus for assessment of diagenesis before isotope analysis are still in the exploratory stage. Only Price et al^8^ have previously measured IRSF values in archaeological dental calculus before isotope analysis. The values for C/P ratio and IRSF that indicate that a bone is unaltered are not expected to be relevant to understanding dental calculus preservation quality. Further work is still required to study the FTIR‐ATR spectra in dental calculus and to identify the exact phosphates and carbonate spectra that can be used specifically for dental calculus diagenesis analysis. Therefore, dental calculus parameters are provided only for building a database for dental calculus data that can be used to further analyse calculus structure and its diagenetic alteration (Table S3). The parameters found in this study, however, cannot be compared to those of the study of Price et al^8^ because that study did not use the same FTIR‐ATR methodology.

#### 3.1.1 Infrared splitting factor

The crystallinity (IRSF) values in modern bones ranged from 3.04 to 3.38 (mean = 3.28 ± 0.09; see Table S3; Figure 1A), slightly lower than the value (IRSF = 3.357 ± 0.007) that was obtained for the modern bones in Kontopoulos et al.^71^ The crystallinity in all the archaeological samples in this project range from 3.50 to 4.05 (medieval mean = 3.74 ± 0.12; post‐medieval mean = 3.82 ± 0.17), higher than both the samples in Kontopoulos et al^71^ and the modern bones from this study. Alteration induced by age‐related diagenesis is demonstrated for any bone if its crystallinity is higher than that of the modern samples. The study by Kontopoulos et al^72^ revealed that archaeological samples with IRSF values greater than 4.2 did not preserve DNA; therefore, all archaeological bone samples with values higher than 4.2 were excluded from this study on the assumption that they may have poor bioapatite preservation as well. The crystallinity values for modern dental calculus samples ranged from 3.28 to 3.95 (mean = 3.66 ± 0.19) and those for archaeological samples ranged from 3.14 to 4.02 (medieval mean = 3.71 ± 0.24; post‐medieval mean = 3.55 ± 0.23). The mean IRSF values for all groups in this study are higher than the value (mean = 3.2 ± 0.2) observed by Price et al,^8^ but this could be due to the small sample size (*n* = 7 of 28) they analysed in their study. For the present study, only grains between 20 and 50 μm particle size were used and were sampled using the ATR technique with FTIR. Price et al^8^ did not use ATR nor did they consider the effect of sample particle size on FTIR measurements as observed previously in bones.^48^ There seems to be no clear relationship or a constant offset between each individual's bone and calculus IRSF values (Figure 1A).

#### 3.1.2 Carbonate‐to‐phosphate ratio (C/P)

The modern bone CO_3_/PO_4_ absorbance ratios range from 0.21 to 0.36 (mean = 0.28 ± 0.04) and those for the archaeological samples range from 0.12 to 0.22 (medieval mean = 0.17 ± 0.03; post‐medieval mean = 0.17 ± 0.02) indicating remarkable similarity between the two archaeological sets of samples (Table S3; Figure 1B). The C/P values for all of the archaeological bones fell below the mean C/P values that were obtained from modern bones analysed in this study as well as those that have been previously obtained in modern unaltered bone (mean C/P = 0.24 ± 0.003)^71^ indicating a loss of the carbonate fraction from the bone apatite.

The C/P ratios for modern dental calculus samples ranged from 0.03 to 0.13 (mean = 0.07 ± 0.02) and those for archaeological samples ranged from 0.04 to 0.17 (medieval mean = 0.11 ± 0.03; post‐medieval mean = 0.09 ± 0.03) (Table S3). Similarly, there seems to be no relationship between the individual bone and calculus C/P values (Figure 1B). Hayashizaki et al^9^ revealed that the carbonate content in dental calculus was higher when compared to other biological apatites such as bone, enamel and dentine; therefore, the C/P ratios in dental calculus were expected to be higher in this study. However, the C/P ratios of dental calculus in this study are lower than that of bone, suggesting that there could be other contributing factors. Dental calculus contains non‐apatitic calcium phosphates,^9^ which are not present in other normal mineralised tissues.^73^ Dental calculus may therefore have an inherent higher phosphate content since, in addition to non‐apatitic calcium phosphates, it also has hydroxyapatite leading to a potentially lower C/P ratio relative to bone. The mean value of specific phosphorus concentration in human rib bone weights has been found to be 8.42 ± 2.14% of dry bone weight^74^ whereas that of dental calculus has been found to be 19%.^75^ This strongly suggests that phosphates may be the cause of the lower C/P values in dental calculus when compared to bone.

Moreover, it was also observed that unlike in bone, the most recent calculus samples have the lowest C/P ratio followed by the post‐medieval samples and then finally the medieval samples. This pattern seems to indicate that the C/P ratio of dental calculus increases with the age of the sample. On the other hand, Hayashizaki et al^9^ revealed that carbonate content in dental calculus depended on its location in the mouth such that the lower anterior teeth have higher carbonate content compared to the upper posterior teeth. All the calculus in this study was collected from posterior teeth, but randomly from either the mandible or maxilla. Therefore, if differences in carbonate content can occur due to the location of where calculus was formed,^9^ it is possible that the apparent trend in C/P ratios observed in this study could simply be a function of where each sample was formed (Figure 1B).

#### 3.1.3 IRSF and C/P relationship

Overall, the bone IRSF and C/P ratios display a very strong inverse correlation for modern and post‐medieval bone samples (Figure 2A). The weaker correlation in medieval samples may relate to the varied ages of the burials (7th to 16th century) as well as the different environments from which they were recovered. Environmental factors that degrade samples differ from site to site and, even in the same setting, the type of burial can influence how environmental factors interact with archaeological remains. Moreover, the longer the remains are buried, the greater the diagenetic alteration.^76^, ^77^ Alteration of the carbonate content in skeletal material has also been shown to be site‐specific.^78^, ^79^ Both the modern and post‐medieval populations were each obtained from a single site while the medieval samples were collected from three sites. There is, however, a strong inverse correlation for dental calculus samples in all periods (Figure 2B). Since the relationship is negative for both bones and dental calculus (Figure 2), there is a general trend of increasing IRSF values with a reduction in C/P values reflecting the loss of carbonate with increasing crystallinity. This is in keeping with the previously reported work on bones.^80^

### 3.2 Isotopes

Collagen quality was assessed using the established collagen quality criteria.^81^, ^82^ All samples in this study produced sufficient collagen for mass spectrometry (Table S1), with collagen yields ranging between 2.7% and 24.0% (mean = 14.5%). The atomic C:N values ranged from 3.1 to 3.4 and are within the range of C:N of 2.9 to 3.6 that DeNiro^83^ specified as acceptable. Furthermore, the percentage of carbon and nitrogen in all the collagen samples from bone fell within the elemental percentages reported for modern mammalian bone collagen by Ambrose^81^ that ranged between 15.3% and 47% and between 5.5% and 17.3% by weight for carbon and nitrogen, respectively.

The mean bone collagen δ^13^C values for medieval, post‐medieval and modern populations are −19.7 ± 0.4‰, −19.8 ± 0.5‰ and −14.0 ± 0.6‰ while their mean δ^15^N values are 11.7 ± 0.9‰, 11.8 ± 1.1‰ and 10.8 ± 0.6‰ respectively. In addition, the mean bone carbonate δ^13^C values for the same populations are −13.4 ± 0.8‰, −14.3 ± 1.0‰ and −9.3 ± 1.1‰ while their mean enamel carbonate δ^13^C values are −13.6 ± 1.0‰, −13.3 ± 1.0‰ and −6.8 ± 1.1‰ respectively (Figure S1; Table 1). There are statistically significant differences in both δ^13^C and δ^15^N values between populations as determined by the Kruskal–Wallis H test (bone collagen δ^13^C: *X* ^2^(2) = 38.295, *p* < 0.001; δ^15^N: *X* ^2^(2) = 14.730, *p* = 0.001; bone carbonate δ^13^C: *X* ^2^(2) = 41.149, *p* < 0.001; calculus carbonate δ^13^C: *X* ^2^(2) = 37.615, *p* < 0.001; enamel carbonate δ^13^C: *X* ^2^(2) = 38.489, *p* < 0.001). The *post hoc* comparisons from the Kruskal–Wallis test revealed that for all tissue δ^13^C values and bone collagen δ^15^N values there were significant differences between the modern population and both the medieval and post‐medieval populations. All modern tissues had higher mean δ^13^C values and lower mean δ^15^N values than both the medieval and post‐medieval populations (Table 1). There was, however, no statistically significant difference between the medieval and the post‐medieval populations (Table S5).

**TABLE 1:** Descriptive statistics for all δ^13^C (‰) and δ^15^N (‰) isotope values being investigated in this study. Contains isotope data, produced by the British Geological Survey, UKRI

	Site	*N*	Min.	Max.	Mean ± 1*σ*	Range
Bone δ^13^C_coll_ (‰)	Medieval	22	−20.9	−18.9	−19.7 ± 0.4	1.9
	Post‐medieval	15	−20.7	−19.1	−19.8 ± 0.5	1.6
	Modern	20	−15.3	−12.4	−14.0 ± 0.6	2.9
Bone δ^15^N_coll_ (‰)	Medieval	22	10.1	13.7	11.7 ± 0.9	3.6
	Post‐medieval	15	9.5	13.0	11.8 ± 1.1	3.5
	Modern	20	9.6	12.0	10.8 ± 0.6	2.4
Bone δ^13^C_carb_ (‰)	Medieval	22	−14.8	−12.1	−13.4 ± 0.8	2.7
	Post‐medieval	15	−15.7	−12.6	−14.3 ± 1.0	3.1
	Modern	20	−11.0	−7.1	−9.3 ± 1.1	3.9
Enamel δ^13^C_carb_ (‰)	Medieval	22	−15.2	−12.1	−13.6 ± 1.0	3.1
	Post‐medieval	15	−14.8	−11.9	−13.3 ± 1.0	2.8
	Modern	20	−9.2	−4.6	−6.8 ± 1.1	4.6
Calculus δ^13^C_carb_ (‰)	Medieval	22	−12.7	−8.0	−9.7 ± 1.2	4.7
	Post‐medieval	15	−11.4	−6.1	−9.2 ± 1.4	5.3
	Modern	20	−7.0	−1.2	−4.1 ± 1.8	5.8

#### 3.2.1 Correlations between tissues

**FIGURE 3:**
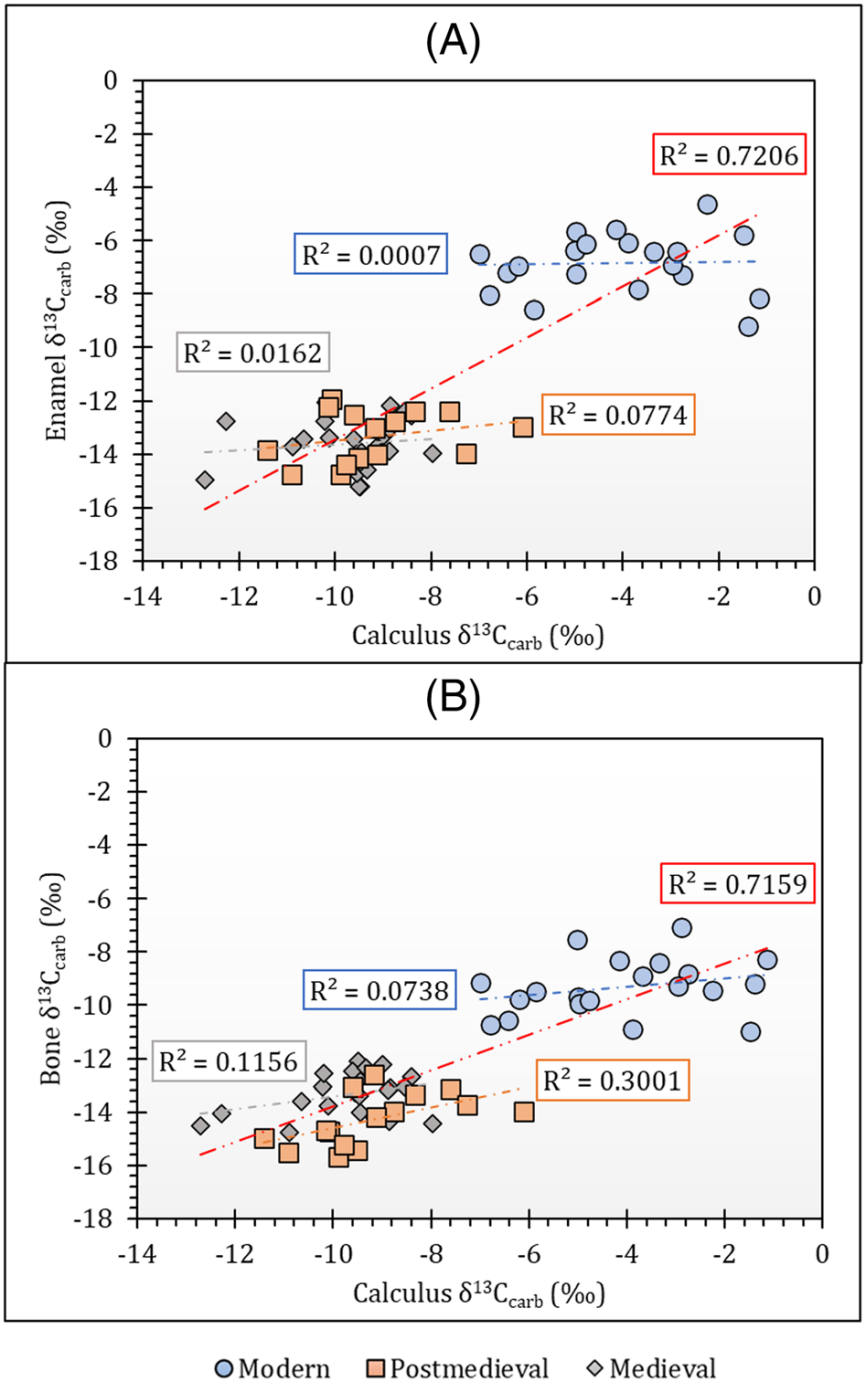
Graphs showing δ^13^C correlations between (A) calculus carbonate and enamel carbonate and (B) calculus carbonate and bone carbonate for separate modern, medieval and post‐medieval population groups. Each trendline is coded according to the symbol colour for each population and the red trendline represents the collective dataset in each graph. Contains isotope data, produced by the British Geological Survey, UKRI [Color figure can be viewed at wileyonlinelibrary.com]

When considered as a collective dataset (the medieval, post‐medieval and modern populations together), there is a strong correlation between bone carbonate δ^13^C values and calculus carbonate δ^13^C values and between enamel δ^13^C values and calculus δ^13^C values (enamel δ^13^C and calculus δ^13^C: *R* ^2^ = 0.7206; bone δ^13^C and calculus δ^13^C: *R* ^2^ = 0.7159; see Figures 3A and 3B). When analysed as discrete populations, however, there is no correlation between either calculus carbonate δ^13^C values and bone carbonate δ^13^C values or calculus carbonate δ^13^C values and enamel carbonate δ^13^C values (Figures 3A and 3B). A similar phenomenon was observed by Eerkens et al^7^ in their analysis of paired calculus and bone carbonates from North American hunter‐gatherers and northeastern African agriculturalists. Eerkens et al^7^ noted that strong correlations are observed when analysing the collective data of individuals consuming distinct diets; however, correlations between bones and calculus within sites were weaker because of a limited range of isotopic values that were found when individuals were consuming similar diets, and this study agrees with that conclusion.

#### 3.2.2 Carbonate δ^13^C tissue offsets

**FIGURE 4:**
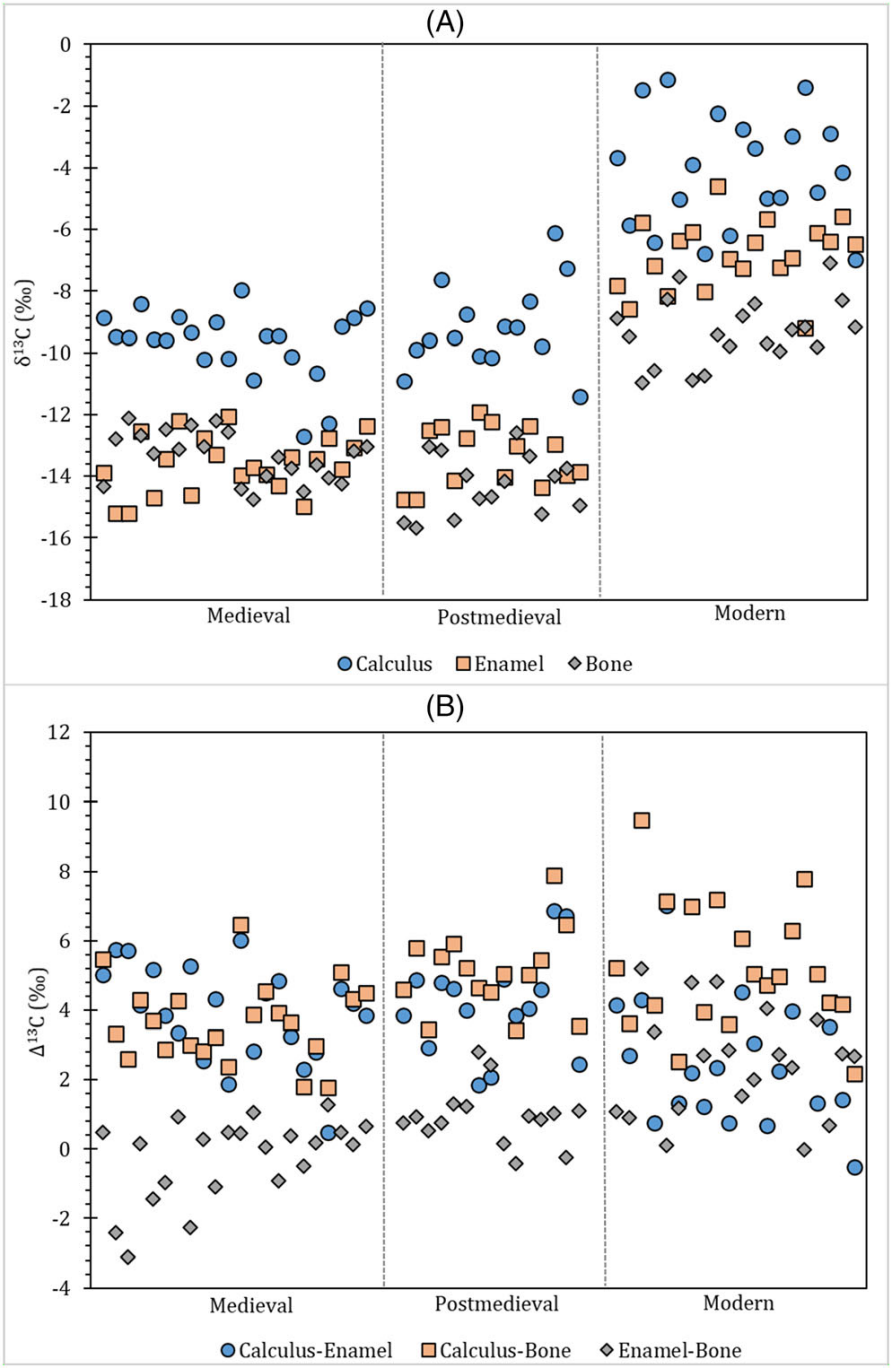
(A) Carbon isotopes by individual data trends and (B) carbon isotope offsets (difference, Δ) by individual data trends. Contains isotope data, produced by the British Geological Survey, UKRI [Color figure can be viewed at wileyonlinelibrary.com]

As expected, the modern population shows higher δ^13^C values in all three tissue carbonates compared to both the medieval and post‐medieval populations. Except for four individuals (FAC 10, FAC 13, FAC 20 and NFC 80), the offset in dental calculus and either bone or enamel carbonate δ^13^C values is usually large and consistent in direction, i.e. except FAC 20, in all populations, dental calculus δ^13^C values are always significantly higher (Figure 4). The modern, medieval and post‐medieval carbonate △^13^C_calculus‐enamel_ and △^13^C_calculus‐bone_ spacing are listed in Table S2. However, there is no consistent offset between the δ^13^C values for the three tissues per individual (Table S2; Figure 4). No constant offsets were observed in Price et al,^8^ the one other study that has also examined calculus mineral and bone carbonate. Despite this, the authors suggested that their average calculus–bone offset of 3.1‰ was probably due to the isotopic exchange between saliva and atmospheric CO_2_.

In contrast to Price et al,^8^ we suggest that a direct comparison between different tissues' isotopic composition is not strictly possible as there may be a difference between the isotopic composition of carbon preserved in bone, teeth and dental calculus. For example, the isotopic composition of carbon in bone carbonate and enamel carbonate has been investigated by Warinner and Tuross,^84^ who compared bone and enamel carbonate of pigs raised on controlled diets containing either raw maize or nixtamalized maize for 13 weeks. The results revealed a significant difference between bone and enamel carbonate in the animals with the δ^13^C values in enamel being higher by 2.2‰ and 2.3‰ in the nixtamalized and raw diets, respectively. Since simultaneously forming bone and enamel carbonate were used, the offset could not be attributed to ‘preferential or differential digestion since the carbonate in both the enamel and bone apatite was deposited from dissolved blood bicarbonate during the same experiment’.^84^ Considering that enamel, once formed, cannot be remodelled, whereas bone undergoes constant remodelling, the authors suggested that the enamel carbonate and bone carbonate of adult animals, therefore, represented ‘temporally segregated isotopic deposition events’. Therefore, since enamel reflects diet at the time of formation (childhood diet) and bone represents an average from the last few years of life,^85^, ^86^ we propose that the inconsistent offsets between enamel and bone are most likely to be due to each individual's different childhood and adult diets.

Additionally, we propose that, unlike bone and enamel, the isotopic fractionation in dental calculus may not be associated simply with metabolism and food sources. In the case of dental calculus, which is formed from a living biofilm, the isotopic fractionation would likely increase with calculus biofilm thickness, similar to what has been observed in other biofilms.^87^ However, it is also notable that in modern individuals the extent of fractionation is lower than in medieval and post‐medieval populations. Although this study does not have the details on the thickness of the dental calculus biofilm, it is proposed here that greater oral hygiene may lead to less well‐established calcified biofilms. Therefore, it is suggested that since the mechanisms involved in the formation of dental calculus, fractionation effects and turnover time in this tissue are different from that of enamel and bone carbonate, a constant offset is not expected between dental calculus carbonate and either bone carbonate or enamel carbonate.

Further, we disagree with the suggestion by Price et al^8^ that the calculus carbonate δ^13^C values were consistently higher than that of bone carbonate due to the exchange with inhaled atmospheric CO_2_ (which has a δ^13^C value of −7‰) and saliva. Although we agree that it is impossible to exclude atmospheric CO_2_ from the sample being analysed, other studies have established that δ^13^C_breath_ values are reflective of ingested food.^88^, ^89^ Therefore, we argue that since the δ^13^C_breath_ values reflect the isotopic signature of the food ingested and if the calculus δ^13^C values are affected by breath CO_2_ then they will reflect the isotope composition of the food consumed. This is, of course, in addition to the bicarbonate derived from saliva that derives carbonate from the blood and the action of microbes when metabolising carbohydrates in the mouth.^30^, ^40^

A final consideration is the potential impact of chemical pre‐treatment steps prior to calculus carbonate isotope analysis. Calculus carbonate is often observed to be enriched in ^13^C compared to bone and enamel carbonate; however, NaClO treatment to remove organics can impact carbon isotope composition as the process induces the adsorption of atmospheric CO_2_ which results in secondary carbonate incorporation.^57^ The subsequent acetic acid treatment carried out here mitigates this by removing such secondary carbonates, but see Pellegrini and Snoeck.^57^ Further work is required to fully understand pre‐treatment effects on dental calculus. However, what is clear is that calculus δ^13^C values track those of bone and enamel in populations analysed here, i.e. calculus δ^13^C values rise in tandem with bone and enamel δ^13^C values in modern versus archaeological populations (Figure 4), suggesting that any impact of pre‐treatment is likely to be minimal.

#### 3.2.3 Dietary interpretation from the three types of tissues

Generally, the carbon isotope values for the modern North American population are extremely high for all tissue types and consistently higher than those of the medieval and post‐medieval populations. Unlike the archaeological populations from England, the modern individuals are influenced by the dominant consumption of C_4_ foods like fructose, maize and its by‐products (e.g. corn syrup).^47^ The medieval and post‐medieval diets, on the other hand, were dominated by C_3_ foods.^17^, ^18^

**FIGURE 5:**
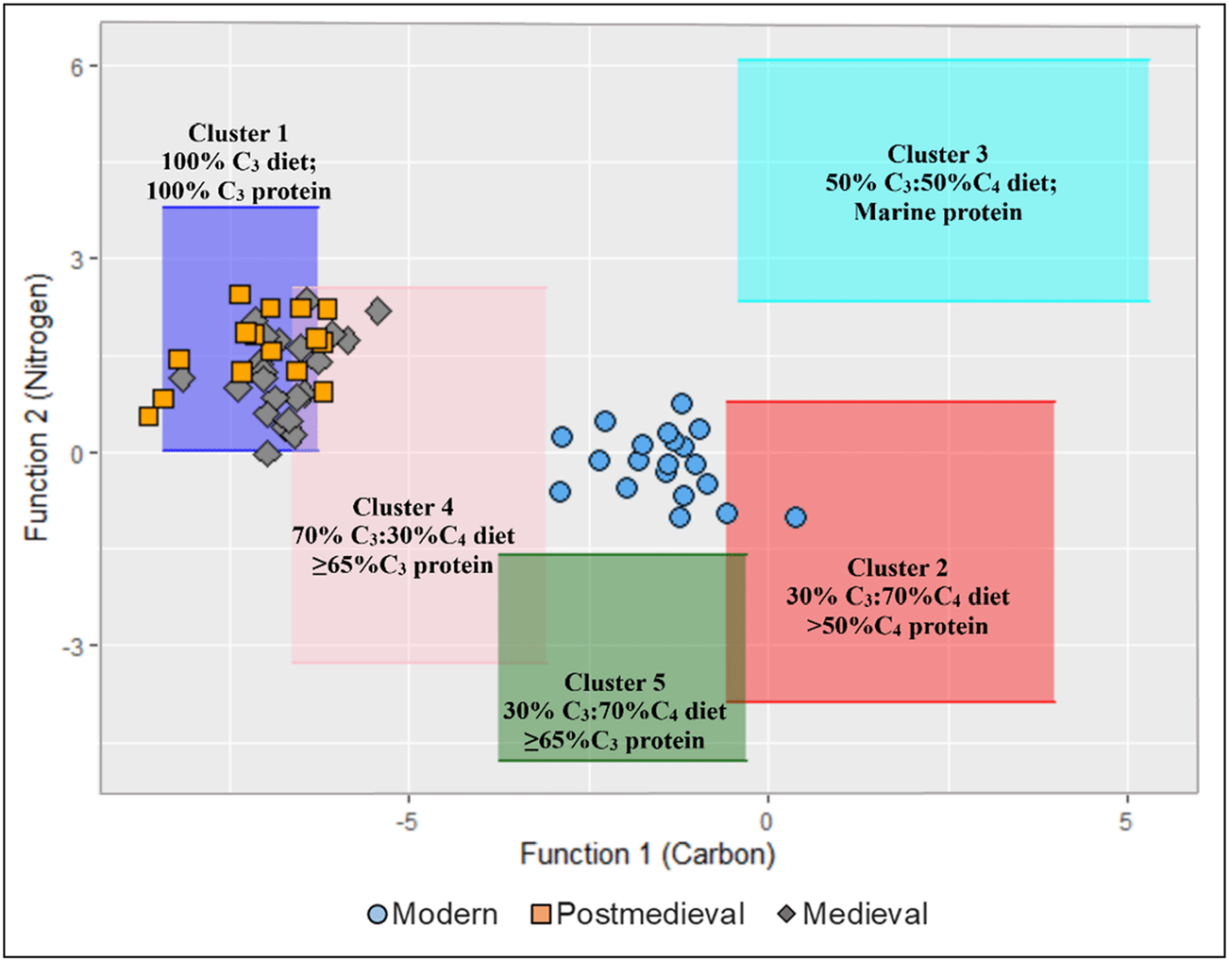
F1 and F2 discriminant function values for all individuals plotted against previously generated dietary clusters (see Froehle et al^90^). Contains isotope data, produced by the British Geological Survey, UKRI [Color figure can be viewed at wileyonlinelibrary.com]

Overall, the mean collagen results for medieval and post‐medieval individuals suggest a diet dominated by C_3_ terrestrial resources while that of the modern individuals is dominated by C_4_ resources as expected. The relatively low δ^15^N values in all populations including the modern individuals suggest that the diets were most likely to have been based on terrestrial rather than marine foods. To further investigate the consumption of C_4_ resources, all individuals were examined using the Froehle et al^90^ multivariate isotope model which incorporates δ^13^C_bone‐collagen_, δ^15^N_bone‐collagen_ and δ^13^C_bone‐carbonate_ data to reconstruct diet. Using published archaeological data, Froehle et al^90^ generated two functions that describe how the test samples varied in terms of isotopic data with the function scores produced from these 2 functions enabling the plotting of data into five discrete clusters of dietary types seen in Figure 5. Seven individuals (SPL 226; SPL 552; SPL 1384; NFC 91; CSM 2.34; CSM 5.09; and CSM 41) in the archaeological population plot within Cluster 4, which corresponds to a total diet of about 30% C_4_ foods and about 35% C_4_ protein in addition to C_3_ foods (Figure 5). An additional 8 individuals (SCN 33; SPL 1063; SPL 1248; NFC 13; NFC 60; CSM 2.31; CSM 5.16; and CSM 37) plot within the area where Clusters 1 and 4 overlap, suggesting that these individuals most likely consumed some C_4_ plants, although it is not clear how much (Figure 5). In contrast, based on the carbon isotope values of enamel, a distinctive C_4_ signature is observed in the modern enamel as expected; however, the C_4_ signature is not so clear with both the medieval and post‐medieval populations. The average δ^13^C values of −13.6 ± 1.0‰ and −13.3 ± 1.0‰ for medieval and post‐medieval samples, respectively, are consistent with the uptake of C_3_ resources. Tooth δ^13^C_enamel_ values are expected to fall between −17‰ and −13‰ for a pure C_3_‐based diet.^91^ The average δ^13^C_enamel_ values for both the medieval and post‐medieval populations are therefore consistent with the typical spectrum of a terrestrial diet in England characterised by high consumption of C_3_ resources as suggested in historical sources.^17^, ^18^

Although there are some suggestions of C_4_ food consumption using the Froehle et al^90^ multivariate isotope model, the use of carbon isotope ratios on dental calculus carbonate should be able to determine if the post‐medieval populations were consuming C_4_ resources (cane sugar/maize). The mean calculus carbonate δ^13^C values for medieval, post‐medieval and modern populations are −9.7 ± 1.2‰, −9.2 ± 1.4‰ and −4.1 ± 1.8‰, respectively. If these δ^13^C values are interpreted as akin to bone carbonate and enamel carbonate values for individuals who consumed an abundance of C_4_ resources (e.g. bone carbonate modern samples for this study = −9.3 ± 1.1‰; enamel carbonate modern samples for this study = −6.8 ± 1.1‰; Tykot et al^11^ study C_4_ bone apatite = −9.8 ± 1.0‰; C_4_ tooth enamel = −8.7 ± 2.3‰), the archaeological populations' calculus δ^13^C values would suggest high inclusion of C_4_ terrestrial resources in their diet. However, the calculus δ^13^C values for archaeological populations are less than those found in modern individuals who in this case are known to have consumed C_4_ foods in large quantities. It should be noted, however, there are slightly higher dental calculus δ^13^C values in some post‐medieval individuals that suggest consumption of a more ^13^C‐enriched carbohydrate such as cane sugar/maize.

There are statistically significant differences in δ^13^C_calculus‐enamel_, δ^13^C_calculus‐bone_ and δ^13^C_enamel‐bone_ values between populations as determined by the Kruskal–Wallis H test (δ^13^C_calculus‐enamel_: *X* ^2^(2) = 8.199, *p* = 0.017; δ^13^C_calculus‐bone_: *X* ^2^(2) = 12.43, *p* = 0.001; δ^13^C_enamel‐bone_: *X* ^2^(2) = 27.24, *p* < 0.001). The *post hoc* comparisons from the Kruskal–Wallis test (Table 2) revealed no significant differences between the modern and post‐medieval populations but significant differences between the medieval and post‐medieval populations in the calculus–bone offsets. The similarity between the post‐medieval data and the modern data supports that the former was consuming some C_4_ foods; however, this similarity is less clear cut as the post‐medieval population was consuming a C_3_ diet with mixed C_3_/C_4_ simple carbohydrates, whereas the modern diet is mixed C_3_/C_4_ with predominantly C_4_ simple carbohydrates. On the other hand, the differences observed between the medieval and post‐medieval populations suggest that there was no input of C_4_ carbohydrate consumption in the medieval population.

**TABLE 2:** *P*‐values for the *post hoc* δ^13^C differences for all populations. The significance level is 0.05 and the significance values have been adjusted by the Bonferroni correction for multiple tests. Contains isotope data, produced by the British Geological Survey, UKRI

Sample 1–sample 2	Calculus–enamel δ^13^C	Calculus–bone δ^13^C	Enamel–bone δ^13^C
Modern–post‐medieval	0.015	0.796	0.030
Modern–medieval	0.014	0.000	0.000
Post‐medieval–medieval	0.847	0.002	0.010

The causes for the differences between bone and enamel are explained in greater detail below.

#### 3.2.4 Causes of diet differences between bone and enamel

The modern individuals' enamel carbonate δ^13^C values for this study are consistently enriched in ^13^C relative to bone carbonate, whereas enamel and bone carbonate for medieval and post‐medieval individuals are broadly similar. Throughout an individual's life bone is constantly remodelled and reflects diet ingested over a number of years prior to death, depending on the skeletal element.^85^ In contrast, enamel in teeth forms over a relatively short time with the materials used in the present study (M1, M2 and M3) completing formation at age 2.5–3, 7–8 and 12–16 years, respectively.^86^ Consequently, the differences observed between these two tissues in modern individuals would be heavily influenced by changes in diet due to age. The enamel carbonate δ^13^C values for the rest of the population are consistent with the well‐documented consumption of sugary foods among children and adolescents.^92^ The total daily energy from added sugars for the American adolescent and teenage population group has been found to be higher than that of adults – approximately 13.1% to 17.5% among children compared to 11.2% to 14.5% among adults.^92^, ^93^ Therefore, Americans consuming more sugar during childhood could create significant offsets between bone and tooth δ^13^C values.

In the case of the medieval and the post‐medieval individuals, the enamel and the bone δ^13^C values are the same because not only are the bones and enamel tapping from the same pool of bicarbonates in the blood during formation, but also the children were not exposed to sugary foods, unlike their modern counterparts. Unlike modern individuals, the archaeological children's diet did not differ substantially from their adult diet, at least in terms of consumption of C_4_ resources.

## 4 CONCLUSIONS

In this study, stable isotope analysis of dental calculus was performed alongside bone and enamel from archaeological samples from England and modern samples from the William M. Bass Donated Skeletal Collection at the University of Tennessee, USA. Diagenesis of dental calculus samples was also assessed, and the calculus diagenetic parameters were compared with those of bone. In general, the availability of modern unaltered calculus enabled this study to introduce FTIR parameters that can be used to investigate diagenesis in dental calculus. It is accepted that the diagenetic process of dental calculus is not fully understood. Although we cannot yet demonstrate whether the archaeological dental samples used here were diagenetically altered or not, this study has provided a large body of data to the dental calculus FTIR dataset. Nevertheless, it is believed that the pre‐treatments that were performed before analysis removed the majority of any diagenetically altered carbonate material. In addition, the unaltered dental calculus revealed a similar pattern of higher calculus carbonate δ^13^C values compared to their bone and enamel values.

Previous studies applying isotope analysis to dental calculus to investigate diet have faced several challenges. This study argues that with additional studies, isotopic analysis of dental calculus carbonates could prove to be a potential method to use in the identification of C_4_ resources. This study effectively produced results that show differences in diet between different populations, whereby higher δ^13^C values found in modern calculus samples confirmed that those people were consuming more C_4_ foods than the English archaeological populations. Furthermore, the data are showing that among the English archaeological populations, there is clearly some C_4_ carbohydrates integrated into the post‐medieval diet. Additionally, it has been established that, within individuals, dental calculus δ^13^C values were (mostly) consistently higher than those of enamel or bone; however, the offset (difference, Δ) in δ^13^C values between the three tissues was not consistent. This could be due to differences in diet at the time of tissue formation or carbon fractionation in the mouth versus the body. The calculus δ^13^C values will most certainly be high due to the sugars that are being metabolised in the mouth as well as the effect of more fractionation in this much more poorly biologically controlled environment compared to that of bone and enamel. Using information from previous breath studies, this study has also demonstrated that it is highly unlikely that atmospheric CO_2_ is causing higher δ^13^C values as suggested by a previous study ^8^ as δ^13^C_breath_ values are reflective of the whole diet. We instead argue that calculus carbonate δ^13^C values are highly likely to represent the carbohydrates consumed. In spite of these conclusions, there is an awareness that the formation process and composition of dental calculus are highly variable between individuals, and therefore there are still a number of gaps in knowledge that need to be addressed before its full potential as a viable tool for palaeodietary studies can be realised. For instance, the effect of pre‐treatment methods and the potential issue of the presence of particles (inorganic debris) in dental calculus are still outstanding, and more information is required to understand how the micro‐debris affects the overall isotope values found in dental calculus. We recommend controlled feeding experiments to clearly observe the effect of different carbohydrates on dental calculus carbon isotope ratios.

### PEER REVIEW

The peer review history for this article is available at https://publons.com/publon/10.1002/rcm.9286.

## Supporting information


**Figure S1:** Human δ^13^C and δ^15^N values from modern, post‐medieval and medieval individuals
**Table S1:** Enamel carbonate, calculus carbonate, bone collagen, bone carbonate isotope data and collagen quality indicators for all samples analysed. Contains Isotope Data, produced by the British Geological Survey, UKRI.
**Table S2**: Individual δ^13^C isotope offsets in this study. Contains Isotope Data, produced by the British Geological Survey, UKRI.
**Table S3**: Summary of bone and dental calculus FTIR‐ATR data displaying the average of data measured in triplicate ‐ IRSF: Infrared splitting factor; C/P: carbonate‐to‐phosphate ratio.
**Table S4:** Modern carbon isotope data with required adjustments/corrections. Contains Isotope Data, produced by the British Geological Survey, UKRI.
**Table S5**: Post hoc results for all populations. The significance level is 0.05 and the significance values have been adjusted by the Bonferroni correction for multiple tests. Contains Isotope Data, produced by the British Geological Survey, UKRI

## Data Availability

The data that supports the findings of this study are available in the supplementary material of this article.
