# Supplementary material for: Isotope analysis of human dental calculus δ13CO3 2−: Investigating a potential new proxy for sugar consumption
Source: Rapid Commun Mass Spectrom. 2022 Mar 25;36(11):e9286. doi: 10.1002/rcm.9286 (PMC9286614; doi:10.1002/rcm.9286)
Supplement: Supplementary file 1 — Figure S1: Human δ13C and δ15N values from modern, post‐medieval and medieval individuals Table S1: Enamel carbonate, calculus carbonate, bone collagen, bone carbonate isotope data and collagen quality indicators for all samples analysed. Contains Isotope Data, produced by the British Geological Survey, UKRI. Table S2: Individual δ13C isotope offsets in this study. Contains Isotope Data, produced by the British Geological Survey, UKRI. Table S3: Summary of bone and dental calculus FTIR‐ATR data displaying the average of data measured in triplicate ‐ IRSF: Infrared splitting factor; C/P: carbonate‐to‐phosphate ratio. Table S4: Modern carbon isotope data with required adjustments/corrections. Contains Isotope Data, produced by the British Geological Survey, UKRI. Table S5: Post hoc results for all populations. The significance level is 0.05 and the significance values have been adjusted by the Bonferroni correction for multiple tests. Contains Isotope Data, produced by the British Geological Survey, UKRI [file RCM-36-0-s001.docx]

**SUPPORTING INFORMATION**

**Modern samples storage and ethical approval for research**

The conditions under which the modern individuals were kept after death ensured minimal degradation due to the environment. When an individual dies and donates their body to the Forensic Anthropology Center (FAC), the body is placed outside at the facility to decompose naturally on the ground surface with a loose plastic covering over it to ensure anonymity. Once the body has skeletonised, the remains are recovered and cleaned of any remnant soft tissue with tap water and a toothbrush. The time between recovery and processing may be a few weeks, a year, or even longer for the individuals who died before 2012. However, since 2012, the time between recovery and curation is weeks to months. Once cleaned, the bones are air-dried, individually labelled, and then placed in an acid-free cardboard box, where they are securely stored in the William M. Bass Donated Skeletal Collection in the Department of Anthropology at the University of Tennessee.

Permission to sample and analyse modern human material was requested from the Forensic Anthropology Center (FAC), University of Tennessee, Knoxville’s donated human body collection and it was granted. All body donations were anonymised to protect the identities of the donors. Furthermore, since all modern human tissue stored at the University of York must comply with the Human Tissue Act (2004) as enforced by the Human Tissue Authority (HTA), approval was sought from the NRES Committee Yorkshire & The Humber – Leeds East REC to work and store modern human tissues at the University of York. This was accepted subject to the work being carried out within the guidelines and SOPs of the York Tissue Bank under the HTA license.

Figure S1: Human δ^13^C and δ^15^N values from modern, post-medieval and medieval individuals


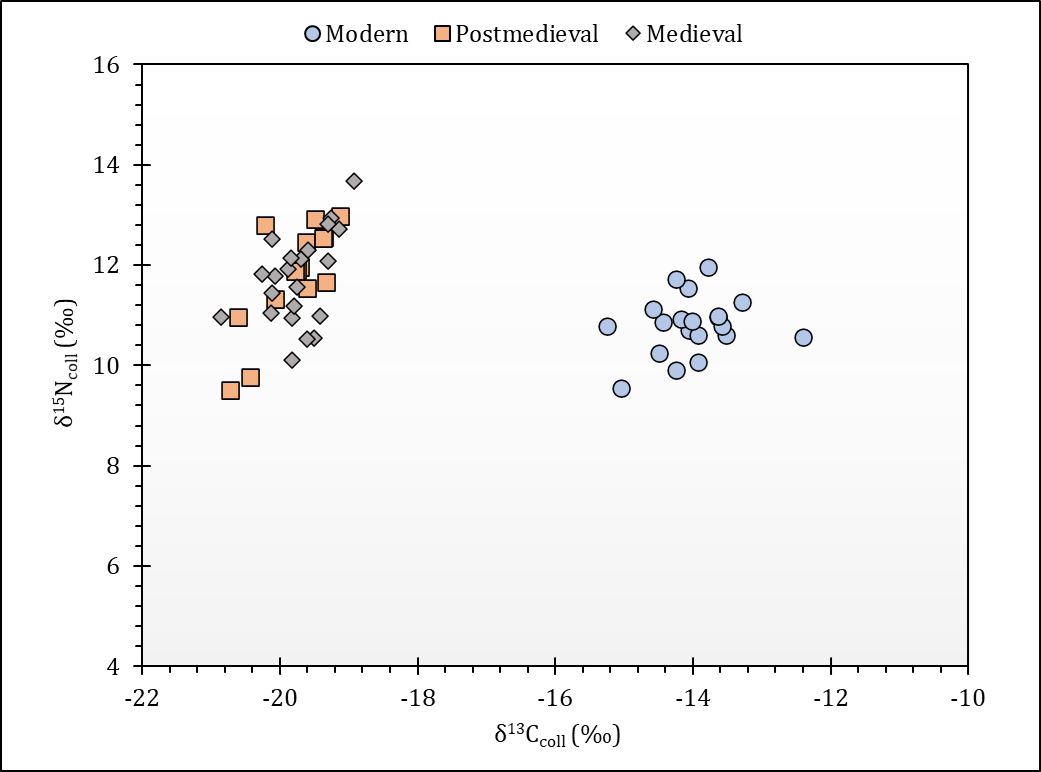


**Table S1****:** Enamel carbonate, calculus carbonate, bone collagen, bone carbonate isotope data and collagen quality indicators for all samples analysed. Contains Isotope Data, produced by the British Geological Survey, UKRI.

| ***Location*** | ***Period*** | ***Sample*** | ***Sex*** | ***Tooth***  ***element*** | ***δ^13^C_calc_ (‰)*** | ***δ^13^C_ena_ (‰)*** | ***Bone***  ***element*** | ***%Coll***  ***yield*** | ***%C*** | ***%N*** | ***At C:N (ratio)*** | ***δ^13^C_coll_ (‰)*** | ***δ^15^N_coll_ (‰)*** | ***δ^13^C_carb_ (‰)*** |
| --- | --- | --- | --- | --- | --- | --- | --- | --- | --- | --- | --- | --- | --- | --- |
| Southwell Cemetery Nottinghamshire | 7^th^ - 9^th^ c. | SCN 20 | M | M2 | -8.9 | -13.9 | Man | 7.6 | 43.9 | 16.3 | 3.1 | -20.1 | 12.5 | -14.3 |
|  | 7^th^ - 9^th^ c. | SCN 27 | F | M1 | -9.5 | -15.2 | Man | 6.4 | 42.9 | 15.8 | 3.2 | -19.8 | 10.1 | -12.8 |
|  | 7^th^ - 9^th^ c | SCN 29 | M | M1 | -9.5 | -15.2 | Man | 3.1 | 42.8 | 15.5 | 3.2 | -20.1 | 11.4 | -12.1 |
|  | 7^th^ - 9^th^ c | SCN 33 | M | M2 | -8.4 | -12.5 | Man | 5.2 | 42.6 | 15.6 | 3.2 | -19.8 | 11.6 | -12.7 |
|  | 7^th^ - 9^th^ c | SCN 35 | M | M1 | -9.6 | -14.7 | Man | 5.3 | 44.0 | 16.3 | 3.2 | -19.8 | 11.2 | -13.3 |
| St Peter's Cemetery Leicester | 10^th^ - 16^th^ c. | SPL 226 | M | M2 | -9.6 | -13.4 | Man | 5.7 | 40.9 | 14.8 | 3.2 | -18.9 | 13.7 | -12.5 |
|  | 10^th^ - 16^th^ c. | SPL 552 | F | M1 | -8.8 | -12.2 | Skull | 10.5 | 41.0 | 15.0 | 3.2 | -19.3 | 12.1 | -13.1 |
|  | 10^th^ - 16^th^ c. | SPL 1063 | F | M1 | -9.3 | -14.6 | Man | 14.7 | 41.9 | 15.3 | 3.2 | -19.9 | 11.9 | -12.3 |
|  | 10^th^ - 16^th^ c. | SPL 1069 | U | M1 | -10.2 | -12.8 | Man | 10.3 | 41.7 | 15.3 | 3.2 | -19.5 | 10.5 | -13.0 |
|  | 10^th^ - 16^th^ c. | SPL 1248 | U | M2 | -9.0 | -13.3 | Man | 2.7 | 38.5 | 13.6 | 3.3 | -19.8 | 11.0 | -12.2 |
|  | 10^th^ - 16^th^ c. | SPL 1384 | U | M2 | -10.2 | -12.1 | Man | 11.5 | 39.7 | 14.5 | 3.2 | -19.3 | 12.9 | -12.6 |
| Nun's Field Chester | 12^th^ - 16^th^ c. | NFC 13 | U | M2 | -8.0 | -14.0 | Rib | 19.0 | 41.9 | 15.3 | 3.2 | -19.1 | 12.7 | -14.4 |
|  | 12^th^ - 16^th^ c. | NFC 19 | F | M2 | -10.9 | -13.7 | Rib | 18.9 | 40.4 | 14.8 | 3.2 | -20.9 | 11.0 | -14.8 |
|  | 12^th^ - 16^th^ c. | NFC 39 | F | M2 | -9.5 | -13.9 | Rib | 9.8 | 41.8 | 15.2 | 3.2 | -19.7 | 12.1 | -14.0 |
|  | 12^th^ - 16^th^ c. | NFC 60 | F | M2 | -9.5 | -14.3 | Rib | 7.0 | 42.0 | 15.0 | 3.3 | -19.6 | 12.3 | -13.4 |
|  | 12^th^ - 16^th^ c. | NFC 68 | F | M2 | -10.1 | -13.4 | Rib | 13.5 | 38.9 | 14.4 | 3.2 | -20.1 | 11.8 | -13.8 |
|  | 12^th^ - 16^th^ c. | NFC 72 | F | M1 | -12.7 | -15.0 | Rib | 5.7 | 30.2 | 11.4 | 3.1 | -19.4 | 11.0 | -14.5 |
|  | 12^th^ - 16^th^ c. | NFC 74 | F | M2 | -10.7 | -13.4 | Rib | 7.3 | 30.7 | 11.5 | 3.1 | -19.6 | 10.5 | -13.6 |
|  | 12^th^ - 16^th^ c. | NFC 80 | F | M2 | -12.3 | -12.8 | Rib | 8.4 | 31.2 | 11.4 | 3.2 | -20.1 | 11.0 | -14.1 |
|  | 12^th^ - 16^th^ c. | NFC 81 | F | M2 | -9.2 | -13.8 | Rib | 13.6 | 38.0 | 14.0 | 3.2 | -19.8 | 12.1 | -14.2 |
|  | 12^th^ - 16^th^ c. | NFC 86 | M | M2 | -8.9 | -13.1 | Rib | 9.2 | 36.6 | 12.6 | 3.4 | -20.3 | 11.8 | -13.2 |
|  | 12^th^ - 16^th^ c. | NFC 91 | M | M2 | -8.5 | -12.4 | Rib | 14.6 | 35.1 | 13.0 | 3.2 | -19.3 | 12.8 | -13.0 |
| Cross Street Manchester | 18^th^ - 19^th^ c. | CSM 2.15 | F | M2 | -10.9 | -14.8 | Ribs | 5.9 | 41.8 | 15.3 | 3.2 | -20.7 | 9.5 | -15.5 |
|  | 18^th^ - 19^th^ c. | CSM 2.18 | M | M2 | -9.9 | -14.8 | Clav | 12.6 | 40.2 | 14.8 | 3.2 | -20.4 | 9.8 | -15.7 |
|  | 18^th^ - 19^th^ c. | CSM 2.31 | F | M2 | -9.6 | -12.5 | Rib | 15.0 | 42.4 | 15.4 | 3.2 | -19.7 | 12.0 | -13.1 |
|  | 18^th^ - 19^th^ c. | CSM 2.34 | F | M2 | -7.6 | -12.4 | Rib | 17.1 | 43.4 | 15.8 | 3.2 | -19.4 | 12.6 | -13.2 |
|  | 18^th^ - 19^th^ c. | CSM 2.36 | M | M2 | -9.5 | -14.1 | Rib | 7.6 | 41.5 | 15.0 | 3.2 | -20.6 | 11.0 | -15.4 |
|  | 18^th^ - 19^th^ c. | CSM 2.37 | M | M2 | -8.8 | -12.8 | MC | 12.9 | 43.7 | 15.9 | 3.2 | -19.8 | 11.9 | -14.0 |
|  | 18^th^ - 19^th^ c. | CSM 2.49 | M | M2 | -10.1 | -11.9 | Rib | 11.3 | 41.6 | 15.3 | 3.2 | -19.6 | 12.5 | -14.7 |
|  | 18^th^ - 19^th^ c. | CSM 4.12 | F | M2 | -10.2 | -12.2 | Rib | 31.5 | 41.5 | 15.2 | 3.2 | -19.8 | 11.9 | -14.7 |
|  | 18^th^ - 19^th^ c. | CSM 4.24 | M | M2 | -9.1 | -14.0 | Rib | 18.4 | 44.6 | 16.5 | 3.2 | -20.1 | 11.3 | -14.2 |
|  | 18^th^ - 19^th^ c. | CSM 5.09 | M | M2 | -9.2 | -13.0 | Rib | 8.0 | 42.2 | 15.5 | 3.2 | -19.3 | 11.7 | -12.6 |
|  | 18^th^ - 19^th^ c. | CSM 5.16 | M | M2 | -8.3 | -12.4 | Rib | 12.6 | 43.5 | 15.9 | 3.2 | -19.4 | 12.5 | -13.3 |
|  | 18^th^ - 19^th^ c. | CSM 12 | M | M2 | -9.8 | -14.4 | Rib | 4.4 | 41.0 | 14.9 | 3.2 | -19.6 | 11.6 | -15.2 |
|  | 18^th^ - 19^th^ c. | CSM 37 | F | M2 | -6.1 | -13.0 | Rib | 19.5 | 42.0 | 15.1 | 3.2 | -19.5 | 12.9 | -14.0 |
|  | 18^th^ - 19^th^ c. | CSM 41 | M | M2 | -7.3 | -14.0 | Rib | 3.1 | 36.7 | 13.3 | 3.2 | -19.1 | 13.0 | -13.7 |
|  | 18^th^ - 19^th^ c. | CSM 62.02 | F | M2 | -11.4 | -13.9 | Rib | 19.9 | 43.0 | 15.8 | 3.2 | -20.2 | 12.8 | -15.0 |
| Forensic Anthropology Center Tennessee, USA | 2013 | FAC 1 | F | M2 | -3.7 | -7.8 | PP | 23.0 | 42.2 | 15.5 | 3.2 | -13.6 | 11.0 | -8.9 |
|  | 2013 | FAC 2 | F | M3 | -5.9 | -8.6 | PP | 21.3 | 42.8 | 15.7 | 3.2 | -14.4 | 10.9 | -9.5 |
|  | 2012 | FAC 3 | F | M2 | -1.5 | -5.8 | PP | 22.5 | 42.4 | 15.6 | 3.2 | -14.2 | 9.9 | -11.0 |
|  | 2014 | FAC 4 | F | M2 | -6.4 | -7.2 | Rib | 23.5 | 42.1 | 15.6 | 3.2 | -15.1 | 9.6 | -10.6 |
|  | 2006 | FAC 5 | F | M2 | -1.2 | -8.2 | PP | 21.0 | 43.4 | 16.1 | 3.2 | -14.1 | 10.7 | -8.3 |
|  | 2008 | FAC 6 | F | M1 | -5.0 | -6.4 | PP | 24.0 | 43.0 | 14.8 | 3.4 | -13.5 | 10.6 | -7.5 |
|  | 2005 | FAC 7 | F | M2/M3 | -3.9 | -6.1 | PP | 21.6 | 41.8 | 15.1 | 3.2 | -15.3 | 10.8 | -10.9 |
|  | 2001 | FAC 8 | F | M2 | -6.8 | -8.0 | PP | 20.7 | 42.6 | 15.7 | 3.2 | -14.6 | 11.1 | -10.7 |
|  | 2001 | FAC 9 | F | M1 | -2.3 | -4.6 | PP | 21.6 | 42.9 | 15.2 | 3.3 | -14.5 | 10.3 | -9.4 |
|  | 2002 | FAC 10 | F | M2 | -6.2 | -7.0 | PP | 22.1 | 42.4 | 15.3 | 3.2 | -13.8 | 12.0 | -9.8 |
|  | 2015 | FAC 11 | M | M2 | -2.8 | -7.3 | PP | 20.2 | 41.9 | 15.3 | 3.2 | -14.1 | 11.6 | -8.8 |
|  | 2013 | FAC 12 | M | M2 | -3.4 | -6.4 | PP | 22.3 | 42.8 | 16.0 | 3.1 | -13.6 | 10.8 | -8.4 |
|  | 2011 | FAC 13 | M | M2 | -5.0 | -5.7 | PP | 22.5 | 42.9 | 15.5 | 3.2 | -13.3 | 11.3 | -9.7 |
|  | 2011 | FAC 14 | M | M2 | -5.0 | -7.2 | PP | 21.4 | 42.5 | 15.6 | 3.2 | -14.2 | 10.9 | -10.0 |
|  | 2016 | FAC 15 | M | M3 | -3.0 | -6.9 | HP | 21.4 | 43.6 | 16.1 | 3.2 | -13.9 | 10.6 | -9.3 |
|  | 2010 | FAC 16 | M | M2 | -1.4 | -9.2 | PP | 21.3 | 43.2 | 16.1 | 3.1 | -14.0 | 10.9 | -9.2 |
|  | 2000 | FAC 17 | M | M2 | -4.8 | -6.1 | Rib | 22.1 | 41.7 | 15.7 | 3.1 | -13.6 | 11.0 | -9.8 |
|  | 1999 | FAC 18 | M | M3 | -2.9 | -6.4 | PP | 22.9 | 42.7 | 15.7 | 3.2 | -12.4 | 10.6 | -7.1 |
|  | 1996 | FAC 19 | M | M2 | -4.2 | -5.6 | PP | 22.2 | 42.9 | 15.6 | 3.2 | -13.9 | 10.1 | -8.3 |
|  | 1997 | FAC 20 | M | M2 | -7.0 | -6.5 | PP | 22.0 | 42.1 | 15.1 | 3.3 | -14.3 | 11.7 | -9.2 |

**Sex category**: U =Sex not determined, F = Female, M =Male

**Age category**: All adults

**Bone Element**: Man=Mandible; Clav=Clavicle; MC=Metacarpal; PP=Pedal phalanx; HP=Hand phalanx

**Heading**: δ^13^C_calc_ = δ^13^Ccalculus; δ^13^C_ena_ = δ^13^Cenamel; δ^13^C_coll_= δ^13^Ccollagen; δ^13^C_carb_ = δ^13^Ccarbonate

**Table S2**: Individual δ^13^C isotope offsets in this study. Contains Isotope Data, produced by the British Geological Survey, UKRI.

| **Period** | **Sample** | **Calculus δ^13^C (‰)** | **Enamel δ^13^C (‰)** | **Bone δ^13^C (‰)** | **△^13^C_calculus-enamel_** | **△^13^C_calculus-bone_** | **△^13^C_enamel-bone_** |
| --- | --- | --- | --- | --- | --- | --- | --- |
| Medieval | SCN 20 | -8.9 | -13.9 | -14.3 | 5.0 | 5.5 | 0.5 |
|  | SCN 27 | -9.5 | -15.2 | -12.8 | 5.7 | 3.3 | -2.4 |
|  | SCN 29 | -9.5 | -15.2 | -12.1 | 5.7 | 2.6 | -3.1 |
|  | SCN 33 | -8.4 | -12.5 | -12.7 | 4.1 | 4.3 | 0.2 |
|  | SCN 35 | -9.6 | -14.7 | -13.3 | 5.2 | 3.7 | -1.5 |
|  | SPL 226 | -9.6 | -13.4 | -12.5 | 3.8 | 2.9 | -1.0 |
|  | SPL 552 | -8.8 | -12.2 | -13.1 | 3.4 | 4.3 | 0.9 |
|  | SPL 1063 | -9.3 | -14.6 | -12.3 | 5.3 | 3.0 | -2.3 |
|  | SPL 1069 | -10.2 | -12.8 | -13.0 | 2.6 | 2.8 | 0.3 |
|  | SPL 1248 | -9.0 | -13.3 | -12.2 | 4.3 | 3.2 | -1.1 |
|  | SPL 1384 | -10.2 | -12.1 | -12.6 | 1.9 | 2.4 | 0.5 |
|  | NFC 13 | -8.0 | -14.0 | -14.4 | 6.0 | 6.5 | 0.5 |
|  | NFC 19 | -10.9 | -13.7 | -14.8 | 2.8 | 3.9 | 1.1 |
|  | NFC 39 | -9.5 | -13.9 | -14.0 | 4.5 | 4.6 | 0.1 |
|  | NFC 60 | -9.5 | -14.3 | -13.4 | 4.9 | 3.9 | -0.9 |
|  | NFC 68 | -10.1 | -13.4 | -13.8 | 3.3 | 3.6 | 0.4 |
|  | NFC 72 | -12.7 | -15.0 | -14.5 | 2.3 | 1.8 | -0.5 |
|  | NFC 74 | -10.7 | -13.4 | -13.6 | 2.8 | 3.0 | 0.2 |
|  | NFC 80 | -12.3 | -12.8 | -14.1 | 0.5 | 1.8 | 1.3 |
|  | NFC 81 | -9.2 | -13.8 | -14.2 | 4.6 | 5.1 | 0.5 |
|  | NFC 86 | -8.9 | -13.1 | -13.2 | 4.2 | 4.3 | 0.1 |
|  | NFC 91 | -8.5 | -12.4 | -13.0 | 3.8 | 4.5 | 0.7 |
| Post-medieval | CSM 2.15 | -10.9 | -14.8 | -15.5 | 3.8 | 4.6 | 0.8 |
|  | CSM 2.18 | -9.9 | -14.8 | -15.7 | 4.9 | 5.8 | 0.9 |
|  | CSM 2.31 | -9.6 | -12.5 | -13.1 | 2.9 | 3.5 | 0.5 |
|  | CSM 2.34 | -7.6 | -12.4 | -13.2 | 4.8 | 5.5 | 0.8 |
|  | CSM 2.36 | -9.5 | -14.1 | -15.4 | 4.6 | 5.9 | 1.3 |
|  | CSM 2.37 | -8.8 | -12.8 | -14.0 | 4.0 | 5.2 | 1.2 |
|  | CSM 2.49 | -10.1 | -11.9 | -14.7 | 1.9 | 4.7 | 2.8 |
|  | CSM 4.12 | -10.2 | -12.2 | -14.7 | 2.1 | 4.5 | 2.4 |
|  | CSM 4.24 | -9.1 | -14.0 | -14.2 | 4.9 | 5.0 | 0.2 |
|  | CSM 5.09 | -9.2 | -13.0 | -12.6 | 3.9 | 3.4 | -0.4 |
|  | CSM 5.16 | -8.3 | -12.4 | -13.3 | 4.0 | 5.0 | 1.0 |
|  | CSM 12 | -9.8 | -14.4 | -15.2 | 4.6 | 5.5 | 0.9 |
|  | CSM 37 | -6.1 | -13.0 | -14.0 | 6.9 | 7.9 | 1.0 |
|  | CSM 41 | -7.3 | -14.0 | -13.7 | 6.7 | 6.5 | -0.2 |
|  | CSM 62.02 | -11.4 | -13.9 | -15.0 | 2.5 | 3.6 | 1.1 |
| Modern | FAC 01 | -3.7 | -7.8 | -8.9 | 4.2 | 5.2 | 1.1 |
|  | FAC 02 | -5.9 | -8.6 | -9.5 | 2.7 | 3.6 | 0.9 |
|  | FAC 03 | -1.5 | -5.8 | -11.0 | 4.3 | 9.5 | 5.2 |
|  | FAC 04 | -6.4 | -7.2 | -10.6 | 0.8 | 4.1 | 3.4 |
|  | FAC 05 | -1.2 | -8.2 | -8.3 | 7.0 | 7.1 | 0.1 |
|  | FAC 06 | -5.0 | -6.4 | -7.5 | 1.3 | 2.5 | 1.2 |
|  | FAC 07 | -3.9 | -6.1 | -10.9 | 2.2 | 7.0 | 4.8 |
|  | FAC 08 | -6.8 | -8.0 | -10.7 | 1.2 | 3.9 | 2.7 |
|  | FAC 09 | -2.3 | -4.6 | -9.4 | 2.4 | 7.2 | 4.8 |
|  | FAC 10 | -6.2 | -7.0 | -9.8 | 0.8 | 3.6 | 2.8 |
|  | FAC 11 | -2.8 | -7.3 | -8.8 | 4.5 | 6.1 | 1.5 |
|  | FAC 12 | -3.4 | -6.4 | -8.4 | 3.1 | 5.1 | 2.0 |
|  | FAC 13 | -5.0 | -5.7 | -9.7 | 0.7 | 4.7 | 4.0 |
|  | FAC 14 | -5.0 | -7.2 | -10.0 | 2.3 | 5.0 | 2.7 |
|  | FAC 15 | -3.0 | -6.9 | -9.3 | 4.0 | 6.3 | 2.3 |
|  | FAC 16 | -1.4 | -9.2 | -9.2 | 7.8 | 7.8 | 0.0 |
|  | FAC 17 | -4.8 | -6.1 | -9.8 | 1.3 | 5.0 | 3.7 |
|  | FAC 18 | -2.9 | -6.4 | -7.1 | 3.5 | 4.2 | 0.7 |
|  | FAC 19 | -4.2 | -5.6 | -8.3 | 1.4 | 4.2 | 2.7 |
|  | FAC 20 | -7.0 | -6.5 | -9.2 | -0.5 | 2.2 | 2.7 |

**Table S3**: Summary of bone and dental calculus FTIR-ATR data displaying the average of data measured in triplicate - IRSF: Infrared splitting factor; C/P: carbonate-to-phosphate ratio.

| **Period** | **Sample** | **Bone IRSF** | **Bone C/P** | **Calculus IRSF** | **Calculus C/P** |
| --- | --- | --- | --- | --- | --- |
| Medieval | SCN 20 | 3.67 ± 0.10 | 0.22 ± 0.00 | 3.26 ± 0.03 | 0.17 ± 0.00 |
|  | SCN 27 | 3.59 ± 0.01 | 0.17 ± 0.00 | 3.36 ± 0.02 | 0.14 ± 0.00 |
|  | SCN 29 | 3.52 ± 0.02 | 0.16 ± 0.00 | 3.35 ± 0.07 | 0.15 ± 0.01 |
|  | SCN 33 | 3.68 ± 0.05 | 0.20 ± 0.00 | - | - |
|  | SCN 35 | 3.87 ± 0.02 | 0.20 ± 0.00 | 3.28 ± 0.05 | 0.17 ± 0.00 |
|  | SPL 226 | 4.01 ± 0.04 | 0.12 ± 0.00 | 3.70 ± 0.05 | 0.12 ± 0.00 |
|  | SPL 552 | 3.82 ± 0.10 | 0.17 ± 0.02 | 3.90 ± 0.16 | 0.09 ± 0.00 |
|  | SPL 1063 | 3.59 ± 0.01 | 0.19 ± 0.00 | 3.71 ± 0.05 | 0.12 ± 0.00 |
|  | SPL 1069 | 3.76 ± 0.00 | 0.17 ± 0.00 | 4.02 ± 0.01 | 0.09 ± 0.00 |
|  | SPL 1248 | 3.74 ± 0.01 | 0.15 ± 0.00 | 3.97 ± 0.07 | 0.09 ± 0.00 |
|  | SPL 1384 | 3.64 ± 0.01 | 0.19 ± 0.00 | 3.97 ± 0.03 | 0.09 ± 0.00 |
|  | NFC 13 | 3.89 ± 0.02 | 0.15 ± 0.00 | 3.93 ± 0.04 | 0.09 ± 0.00 |
|  | NFC 19 | 3.85 ± 0.01 | 0.15 ± 0.00 | 3.83 ± 0.04 | 0.10 ± 0.00 |
|  | NFC 39 | 3.75 ± 0.05 | 0.13 ± 0.00 | 3.87 ± 0.04 | 0.10 ± 0.00 |
|  | NFC 60 | 3.71 ± 0.02 | 0.15 ± 0.00 | 3.69 ± 0.07 | 0.11 ± 0.02 |
|  | NFC 68 | 3.72 ± 0.07 | 0.15 ± 0.00 | 3.81 ± 0.06 | 0.10 ± 0.00 |
|  | NFC 72 | 3.67 ± 0.00 | 0.20 ± 0.00 | 3.86 ± 0.03 | 0.10 ± 0.00 |
|  | NFC 74 | 3.86 ± 0.01 | 0.15 ± 0.00 | 3.87 ± 0.09 | 0.10 ± 0.00 |
|  | NFC 80 | 3.66 ± 0.01 | 0.20 ± 0.00 | - | - |
|  | NFC 81 | 3.64 ± 0.06 | 0.16 ± 0.00 | 3.49 ± 0.09 | 0.12 ± 0.01 |
|  | NFC 86 | 3.70 ± 0.00 | 0.18 ± 0.00 | 3.56 ± 0.02 | 0.12 ± 0.00 |
|  | NFC 91 | 3.92 ± 0.01 | 0.15 ± 0.00 | - | - |
| Post-medieval | CSM 2.15 | 3.68 ± 0.06 | 0.18 ± 0.01 | 3.46 ± 0.04 | 0.09 ± 0.00 |
|  | CSM 2.18 | 3.80 ± 0.02 | 0.17 ± 0.00 | 3.46 ± 0.01 | 0.09 ± 0.00 |
|  | CSM 2.31 | 3.58 ± 0.17 | 0.20 ± 0.03 | 3.48 ± 0.02 | 0.10 ± 0.01 |
|  | CSM 2.34 | 4.05 ± 0.09 | 0.15 ± 0.01 | 3.31 ± 0.13 | 0.13 ± 0.00 |
|  | CSM 2.36 | 4.01 ± 0.08 | 0.14 ± 0.00 | 3.35 ± 0.09 | 0.12 ± 0.00 |
|  | CSM 2.37 | 4.01 ± 0.04 | 0.14 ± 0.00 | 3.14 ± 0.10 | 0.16 ± 0.00 |
|  | CSM 2.49 | 3.87 ± 0.03 | 0.16 ± 0.00 | 3.55 ± 0.08 | 0.10 ± 0.01 |
|  | CSM 4.12 | 3.81 ± 0.02 | 0.16 ± 0.01 | 3.56 ± 0.05 | 0.10 ± 0.01 |
|  | CSM 4.24 | 4.02 ± 0.02 | 0.15 ± 0.00 | 3.23 ± 0.06 | 0.15 ± 0.00 |
|  | CSM 5.09 | 3.60 ± 0.01 | 0.18 ± 0.00 | 3.86 ± 0.01 | 0.08 ± 0.00 |
|  | CSM 5.16 | 3.80 ± 0.02 | 0.18 ± 0.01 | 3.68 ± 0.01 | 0.09 ± 0.00 |
|  | CSM 12 | 3.87 ± 0.04 | 0.16 ± 0.00 | 3.82 ± 0.02 | 0.04 ± 0.00 |
|  | CSM 37 | 3.50 ± 0.04 | 0.21 ± 0.02 | 3.72 ± 0.02 | 0.05 ± 0.00 |
|  | CSM 41 | 3.80 ± 0.00 | 0.18 ± 0.00 | 3.79 ± 0.01 | 0.05 ± 0.00 |
|  | CSM 62.02 | 3.86 ± 0.01 | 0.16 ± 0.00 | 3.83 ± 0.02 | 0.05 ± 0.00 |
| Modern | FAC 01 | 3.21 ± 0.06 | 0.32 ± 0.02 | 3.59 ± 0.05 | 0.08 ± 0.00 |
|  | FAC 02 | 3.36 ± 0.03 | 0.24 ± 0.01 | 3.65 ± 0.01 | 0.10 ± 0.00 |
|  | FAC 03 | 3.19 ± 0.03 | 0.33 ± 0.00 | 3.77 ± 0.09 | 0.05 ± 0.00 |
|  | FAC 04 | 3.22 ± 0.10 | 0.32 ± 0.00 | 3.75 ± 0.06 | 0.07 ± 0.00 |
|  | FAC 05 | 3.38 ± 0.11 | 0.21 ± 0.01 | 3.59 ± 0.00 | 0.08 ± 0.00 |
|  | FAC 06 | 3.16 ± 0.06 | 0.34 ± 0.01 | 3.58 ± 0.03 | 0.06 ± 0.01 |
|  | FAC 07 | 3.35 ± 0.01 | 0.25 ± 0.01 | 3.63 ± 0.07 | 0.08 ± 0.00 |
|  | FAC 08 | 3.28 ± 0.01 | 0.31 ± 0.00 | 3.73 ± 0.05 | 0.07 ± 0.01 |
|  | FAC 09 | 3.37 ± 0.02 | 0.23 ± 0.01 | 3.33 ± 0.03 | 0.09 ± 0.00 |
|  | FAC 10 | 3.35 ± 0.01 | 0.24 ± 0.01 | 3.78 ± 0.02 | 0.07 ± 0.00 |
|  | FAC 11 | 3.16 ± 0.02 | 0.33 ± 0.00 | 3.72 ± 0.04 | 0.07 ± 0.00 |
|  | FAC 12 | 3.32 ± 0.04 | 0.29 ± 0.02 | 3.91 ± 0.07 | 0.03 ± 0.00 |
|  | FAC 13 | 3.34 ± 0.01 | 0.26 ± 0.01 | 3.95 ± 0.01 | 0.04 ± 0.00 |
|  | FAC 14 | 3.33 ± 0.06 | 0.28 ± 0.01 | 3.71 ± 0.05 | 0.07 ± 0.00 |
|  | FAC 15 | 3.04 ± 0.04 | 0.36 ± 0.00 | 3.73 ± 0.12 | 0.05 ± 0.00 |
|  | FAC 16 | 3.35 ± 0.03 | 0.25 ± 0.00 | - | - |
|  | FAC 17 | 3.33 ± 0.12 | 0.27 ± 0.00 | 3.28 ± 0.02 | 0.11 ± 0.00 |
|  | FAC 18 | 3.34 ± 0.05 | 0.26 ± 0.01 | - | - |
|  | FAC 19 | 3.28 ± 0.04 | 0.30 ± 0.00 | 3.84 ± 0.06 | 0.05 ± 0.00 |
|  | FAC 20 | 3.31 ± 0.03 | 0.29 ± 0.00 | 3.28 ± 0.03 | 0.13 ± 0.01 |

**Table S4:** Modern carbon isotope data with required adjustments/corrections. Contains Isotope Data, produced by the British Geological Survey, UKRI.

|  |  |  |  |  |  |  | δ^13^C ‰ values with fossil fuel correction as appropriate^[65]^ | | | |
| --- | --- | --- | --- | --- | --- | --- | --- | --- | --- | --- |
| Sample | Year of Death | δ^13^C_coll_ ‰ | δ^13^C_carb_ ‰ | δ^13^C_calc_ ‰ | δ^13^C_ena_ ‰ | Fossil fuel correction to 1860 | δ^13^C_coll_ ‰ | δ^13^C_carb_ ‰ | δ^13^C_calc_ ‰ | δ^13^C_ena_ ‰ |
| FAC 1 | 2013 | -15.41 | -10.68 | -5.46 | -9.61 | 1.78 | -13.63 | -8.90 | -3.68 | -7.8 |
| FAC 2 | 2013 | -16.22 | -11.26 | -7.64 | -10.35 | 1.78 | -14.44 | -9.48 | -5.86 | -8.6 |
| FAC 3 | 2012 | -15.99 | -12.72 | -3.24 | -7.53 | 1.75 | -14.24 | -10.97 | -1.49 | -5.8 |
| FAC 4 | 2014 | -16.86 | -12.37 | -8.23 | -8.99 | 1.81 | -15.05 | -10.56 | -6.42 | -7.2 |
| FAC 5 | 2006 | -15.69 | -9.91 | -2.78 | -9.80 | 1.63 | -14.06 | -8.28 | -1.15 | -8.2 |
| FAC 6 | 2008 | -15.19 | -9.20 | -6.68 | -8.02 | 1.67 | -13.52 | -7.53 | -5.01 | -6.4 |
| FAC 7 | 2005 | -16.85 | -12.48 | -5.48 | -7.69 | 1.6 | -15.25 | -10.88 | -3.88 | -6.1 |
| FAC 8 | 2001 | -16.04 | -12.19 | -8.25 | -9.48 | 1.46 | -14.58 | -10.73 | -6.79 | -8.0 |
| FAC 9 | 2001 | -15.95 | -10.89 | -3.71 | -6.06 | 1.46 | -14.49 | -9.43 | -2.25 | -4.6 |
| FAC 10 | 2002 | -15.28 | -11.29 | -7.69 | -8.45 | 1.5 | -13.78 | -9.79 | -6.19 | -7.0 |
| FAC 11 | 2015 | -15.9 | -10.64 | -4.58 | -9.10 | 1.83 | -14.07 | -8.81 | -2.75 | -7.3 |
| FAC 12 | 2013 | -15.35 | -10.18 | -5.13 | -8.19 | 1.78 | -13.57 | -8.40 | -3.35 | -6.4 |
| FAC 13 | 2011 | -15.01 | -11.42 | -6.71 | -7.38 | 1.72 | -13.29 | -9.70 | -4.99 | -5.7 |
| FAC 14 | 2011 | -15.89 | -11.67 | -6.69 | -8.94 | 1.72 | -14.17 | -9.95 | -4.97 | -7.2 |
| FAC 15 | 2016 | -15.76 | -11.09 | -4.79 | -8.75 | 1.83 | -13.93 | -9.26 | -2.96 | -6.9 |
| FAC 16 | 2010 | -15.71 | -10.88 | -3.09 | -10.89 | 1.7 | -14.01 | -9.18 | -1.39 | -9.2 |
| FAC 17 | 2000 | -15.09 | -11.27 | -6.23 | -7.55 | 1.45 | -13.64 | -9.82 | -4.78 | -6.1 |
| FAC 18 | 1999 | -13.85 | -8.54 | -4.33 | -7.85 | 1.45 | -12.4 | -7.09 | -2.88 | -6.4 |
| FAC 19 | 1996 | -15.26 | -9.65 | -5.49 | -6.91 | 1.34 | -13.92 | -8.31 | -4.15 | -5.6 |
| FAC 20 | 1997 | -15.62 | -10.53 | -8.36 | -7.85 | 1.37 | -14.25 | -9.16 | -6.99 | -6.5 |

**Heading**: δ^13^C_calc_= δ^13^Ccalculus; δ^13^C_ena_ = δ^13^Cenamel; δ^13^C_coll_ = δ^13^Ccollagen; δ^13^C_carb_ = δ^13^Ccarbonate

**Table S5**: Post hoc results for all populations. The significance level is 0.05 and the significance values have been adjusted by the Bonferroni correction for multiple tests. Contains Isotope Data, produced by the British Geological Survey, UKRI

| Sample 1-Sample 2 | Bone collagen  δ^13^C | Bone collagen  δ^15^N | Bone carbonate δ^13^C | Calculus carbonate  δ^13^C | Enamel carbonate  δ^13^C |
| --- | --- | --- | --- | --- | --- |
| Modern-Post-medieval | 0.000 | 0.03 | 0.000 | 0.000 | 0.000 |
| Modern-Medieval | 0.000 | 0.03 | 0.000 | 0.000 | 0.000 |
| Post-medieval-Medieval | 1.000 | 1.000 | 0.270 | 1.000 | 1.000 |
